# Whole genome analysis of toxic Papilionidae butterflies utilizing aristolochic acid, *Pachliopta aristolochiae*, and *Byasa alcinous*

**DOI:** 10.1093/dnares/dsaf038

**Published:** 2025-12-22

**Authors:** Shinya Komata, Rei Kajitani, Takahiro Yamabe, Tasuku Kitamura, Atsushi Toyoda, Tetsuya Kojima, Takehiko Itoh, Haruhiko Fujiwara

**Affiliations:** School of Life Science and Technology, Institute of Science Tokyo, Meguro-ku, Tokyo 152-8550, Japan; School of Life Science and Technology, Institute of Science Tokyo, Meguro-ku, Tokyo 152-8550, Japan; School of Life Science and Technology, Institute of Science Tokyo, Meguro-ku, Tokyo 152-8550, Japan; Department of Integrated Biosciences, Graduate School of Frontier Sciences, The University of Tokyo, Kashiwa, Chiba 277-8562, Japan; Comparative Genomics Laboratory, National Institute of Genetics, Shizuoka 411-8540, Japan; Advanced Genomics Center, National Institute of Genetics, Shizuoka 411-8540, Japan; Department of Integrated Biosciences, Graduate School of Frontier Sciences, The University of Tokyo, Kashiwa, Chiba 277-8562, Japan; School of Life Science and Technology, Institute of Science Tokyo, Meguro-ku, Tokyo 152-8550, Japan; Department of Integrated Biosciences, Graduate School of Frontier Sciences, The University of Tokyo, Kashiwa, Chiba 277-8562, Japan

**Keywords:** aristolochic acid, comparative genomics, RNAi, toxic butterfly, warning colouration

## Abstract

Several species of toxic butterflies are known, including those from the Troidini tribe of the Papilionidae, which accumulate aristolochic acid from their host plants in Aristolochiae. However, the molecular mechanisms involved in utilizing aristolochic acid remain unknown. Toxic butterflies often exhibit warning colouration to signal their toxicity to predators, a complex adaptive trait with toxin utilization. In this study, we sequenced, assembled, and annotated the genomes of 2 toxic Troidini butterflies, *Pachliopta aristolochiae* (312.1 Mb, 13,497 genes) and *Byasa alcinous* (257.6 Mb, 14,669 genes), and conducted comparative genomics to identify genes involved in toxin utilization and warning colouration. Comparative analysis across 11 species revealed 31 gene families significantly expanded and 417 genes under positive selection. Additionally, 442 genes were highly expressed in the red spots on the hindwings of *P. aristolochiae*. The genes shared within these lists may be involved in the formation of the complex adaptive traits of toxin utilization and warning colouration. Functional analysis using RNAi confirmed the involvement of *ebony*, *laccase2*, and *tyrosine hydroxylase* (*TH*) in warning colouration. This research marks a significant starting point in understanding the genetic basis of aristolochic acid utilization and the formation of warning colouration, providing the first list of candidate genes.

## 1. Introduction

In butterflies, certain species are known to incorporate secondary metabolites from host plants during the larval stage (which act as toxins to predators) to aid in predator avoidance.^1^ Heliconius butterflies, for instance, engage in chemical defense by absorbing cyanogenic glycosides from their *Passiflora* host plants to avoid predation.^2^ Similarly, the monarch butterfly (*Danaus plexippus*) is also a toxic species that consumes and accumulates cyanogenic glycosides from milkweed, and a few amino acid substitutions in transmembrane protein, the alpha subunit of the sodium pump, Na^+^/K^+^ -ATPase (ATPα) allow them to utilize the toxic.^3^ These amino acid substitutions in ATPα, which facilitate cyanogenic glycoside utilization, are shared across 3 insect orders—Lepidoptera, Coleoptera, and Hemiptera.^3^ Furthermore, introducing similar mutations in *Drosophila* ATPα has also been shown to confer resistance to cyanogenic glycosides.^4^ However, in the case of toxic swallowtail butterflies that rely on aristolochic acids, their underlying molecular mechanisms remain entirely unknown.

**Fig. 1.**
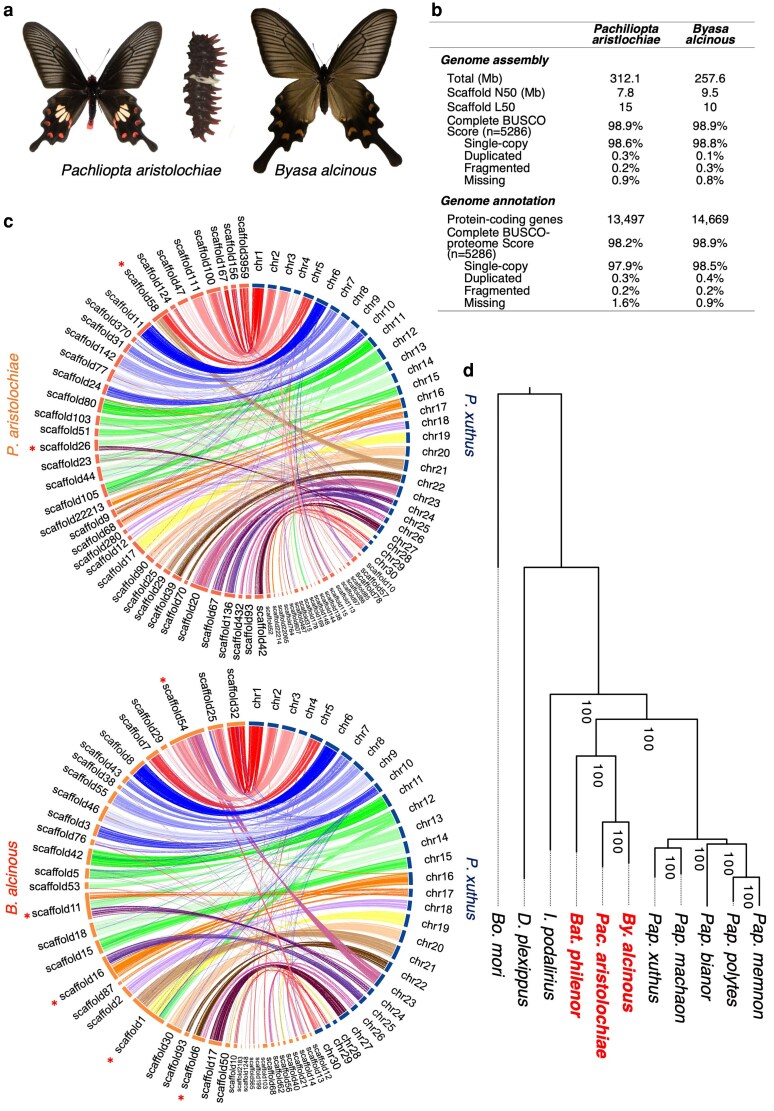
a) Toxic butterflies, *Pachliopta aristolochiae* adult and larvae, and *Byasa alcinous* adult. The larvae have spine-like structures with red tips, serving as a warning colouration to deter predators. b) Summary of genome assembly and gene annotation in 2 toxic butterflies. See Tables S3–S6 for detailed statistics. c) Synteny plots between *P. aristolochiae* and *Papilio xuthus*, and between *B. alcinous* and *P. xuthus. P. xuthus* genome is constructed at the chromosome level (Li *et al*. 2015). Asterisks indicate scaffolds where chromosome fusion may have occurred. d) The maximum likelihood phylogenetic tree of Papilionidae butterflies using single-copy genes from 11 lepidopteran species. The species in red are toxic butterflies that utilize aristolochic acid: *Byasa alcinous, Pachliopta aristolochiae* and *Battus philenor*. The bootstrap values estimated from 1,000 replications are shown near the nodes.

Among swallowtail butterflies that serve as models for Batesian mimicry, such as *Pachliopta aristolochiae* and closely related species like *Byasa alcinous* within the Troidini tribe of the Papilionidae family (Fig. 1a), it is believed that these species evade avian predation by consuming and accumulating aristolochic acid from Aristolochiaceae host plants and that the toxin utilization is associated with warning colouration, with black wings featuring red or yellow spots on the wings and abdomen.^5,6^ Adult *P*. *aristolochiae* have red spots on their black hindwings and red regions along the sides of the abdomen (Fig. 1a). Similarly, *B*. *alcinous* adults have slightly red spots on their wings (with individual variations in redness) and red regions along the abdomen, much like *P*. *aristolochiae* (Fig. 1a). These butterflies also display conspicuous larval forms, featuring structures that appear as spines (though they are soft) (Fig. 1a).^7^ The presence of toxins within the body (making them distasteful as prey) combined with aposematic colouration that signals toxicity to predators suggests an adaptive strategy known as a complex adaptive trait.

The Papilionidae family includes approximately 570 described species, and 3 out of its 8 tribes (Troidini, Luehdorfiini, and Zerynthiini) use Aristolochiaceae plants as host plants.^6^ Luehdorfiini and Zerynthiini are sister tribes and represent a basal lineage within Papilionidae, whereas Troidini is more derived and nested among tribes that do not utilize Aristolochiaceae.^8^ Since these 3 tribes are not monophyletic, it remains debated whether the use of Aristolochiaceae evolved independently across multiple tribes or in a common ancestor of the Papilionidae.^8,9^ Nonetheless, the utilization of Aristolochiaceae (ie ingesting, accumulating, or metabolizing aristolochic acid) is believed to have significantly contributed to the diversification of the Papilionidae family.

Nishida *et al*.^6,10,11^ have conducted extensive research on the use of aristolochic acid and its relationship to life-history traits in *B*. *alcinous* (a synonym for *Atrophaneura alcinous*). In *B*. *alcinous*, aristolochic acid is present in the larvae’s osmeterium, the pupae, the bodies of adults, and even the eggshells laid by females, indicating that *B*. *alcinous* utilize aristolochic acid obtained from their host plants.^10^ There are 7 known aristolochic acid analogs, and liquid chromatography has confirmed that aristolochic acids I and II are particularly abundant in *B*. *alcinous*.^10^ Furthermore, aristolochic acid serves as an oviposition stimulant for *B*. *alcinous* females.^11^ Similarly, in *Battus philenor*—another member of the Troidini tribe—aristolochic acid is found in eggs, larvae, pupae, and adult wings and bodies.^12^ In experiments where *P*. *aristolochiae* was offered to bulbul birds, the birds quickly learned to avoid it.^13^ Aristolochic acid is known to have a bitter taste and nephrotoxic effects when consumed by humans.^6,14^ Additionally, aristolochic acid has been shown to have carcinogenic potential.^15^ Furthermore, the toxicity of aristolochic acid has been documented in *Manduca sexta* (Lepidoptera) and *Anopheles stephensi* (Diptera), indicating that aristolochic acid is toxic not only to predators but also to herbivorous insects.^16,17^ Therefore, the Papilionidae butterflies, especially the Troidini tribe, represent a highly intriguing group due to their unique adaptation to utilize aristolochic acid.

To date, whole-genome sequencing and gene annotation have been completed and published for about 10 species in the Papilionidae butterflies (Table S3). The family Papilionidae includes both Batesian mimics and their model species, such as the toxic butterflies examined in this study. While little is known about the models, the molecular mechanisms underlying Batesian mimicry have been more extensively studied. In *Papilio polytes* and *Papilio memnon*, which mimic Troidini tribe butterflies such as *P*. *aristolochiae*, mimicry is known to occur only in females. The *doublesex* (*dsx*) gene and surrounding regions are involved in the development of mimicry forms, enabling the switch from non-mimetic to mimetic forms.^18–22^ Downstream genes of *dsx* involved in the formation of mimicry wing patterns have also been studied, revealing that the Wnt and Hedgehog signalling pathways play roles in the development of mimicry patterns.^23–25^ From this point forward, elucidating the molecular mechanisms underlying toxin utilization and aposematism in the model species of mimicry will enable a more comprehensive understanding of the molecular basis of both mimicry and models in the Papilionidae. Such insights will further advance our knowledge of the coevolutionary dynamics between models and their mimics.

In this study, we analyzed the whole genomes of *P*. *aristolochiae* and *B*. *alcinous* through de novo assembly and conducted gene annotation. We then performed comparative genomics with other closely related species in the Papilionidae butterflies to identify gene groups with expanded gene counts in these 2 toxic butterflies, as well as to search for genes under positive selection, creating a list of candidate genes potentially involved in aristolochic acid utilization. Additionally, we used RNA-seq to identify genes involved in the formation of warning colouration in the hindwings of *P*. *aristolochiae* and conducted functional analyses through RNA interference (RNAi) via electroporation. Finally, we discuss the candidate genes that may be involved in aristolochic acid metabolism and accumulation as well as in warning colouration formation.

## 2. Methods

### 2.1. Butterfly husbandry

We purchased wild-caught *P*. *aristolochiae* adults from Mr. Y. Irino (Okinawa, Japan) and Mr. I. Aoki (Okinawa, Japan) and obtained eggs and used them for the experiment. *B*. *alcinous* larvae for the experiment were collected in Chiba, Japan. These larvae were fed on the leaves of *Aristolochia debilis* and were kept at 20 to 25 °C under long-day conditions (light:dark = 16:8 h). Adults were fed on a sports drink (Calpis, Asahi. Japan).

### 2.2. De novo assembly and annotation of *P*. *aristolochiae* and *B*. *alcinous*

Genomic DNAs of one female *P*. *aristolochiae* and one male *B*. *alcinous* larvae were extracted from the whole body for whole genome sequencing. In both species, both paired-end and mate-pair (insert sizes, 3, 6, 10, and 15 kb) libraries (Table S1) were prepared and sequencing was performed by Illumina sequencer. Raw Illumina data were trimmed and filtered using Platanus_trim (v1.0.7), and the trimmed data were used for k-mer analysis and estimation of genome size (Figure S1). The draft genomes for *P*. *aristolochiae* and *B*. *alcinous* were assembled with Illumina reads using Platanus (version 1.2.6).^26^ Short scaffolds (length, <500 bp) and contamination candidates that matched bacterial or viral genomes (database, National Center for Biotechnology Information Bacteria and RefSeq viral genomes; tool, Blastn; threshold identity, ≥90%; and query coverage, ≥50%) were excluded. The complete mitochondrial genome was assembled by Novoplasty (v4.2),^27^ and the resulting complete genome was added to the draft genome. The completeness of the genome assembly was evaluated using BUSCO v5.4.3 with the lepidoptera_odb10 database (5,286 genes).^28^

The annotation of protein-coding genes was performed using the combined results of homology-based, RNA-seq-based, and ab initio-based prediction methods according to GINGER pipeline.^29^ Homology-based predictions were performed using the amino acid sequences from following species: *Papilio polytes*, *Bombyx mori*, *Papilio memnon*, *Acyrthosiphon pisum*, *Papilio xuthus*, *Apis mellifera*, *Papilio machaon*, *Drosophila melanogaster*, *Papilio bianor*, *Tribolium castaneum*, *Heliconius melpomene*, *B*. *alcinous* (the amino acid sequence by Trinity *denovo* assembly of RNA-seq was used for the annotation of *P*. *aristolochiae*), *Danaus plexippus*. The sequences were downloaded from the NCBI database. RNA-seq data used in gene annotation are shown in Table S2 (methods for obtaining RNA-seq are described below.). The completeness of the annotated genes was evaluated using BUSCO v5.4.3 (protein mode) with the lepidoptera_odb10 database.^28^

### 2.3. Synteny analysis

We compared synteny between 2 genome assemblies constructed in this study and 2 chromosome-level *Papilio* butterflies assemblies: *P*. *xuthus*^30^ and *P*. *machaon* (NCBI RefSeq: GCF_912999745.1). Alignments were conducted using Minimap2 (v2.26-r1175)^31^ and visualized using circos (v0.69-8).^32^ About 2 Mb or larger scaffolds were targeted, and alignments with a length of 500 bp or more and identity of 70% or more were displayed.

### 2.4. Gene family expansion in toxic butterflies, *P*. *aristolochiae*, *B*. *alcinous,* and *B. philenor*

Using 11 lepidopteran species (Table S3), orthologous gene groups (orthogroups) were predicted using Sonicparanoid (v2.0.4).^33^ The 11 species included 3 that utilize aristolochic acid (*Pachliopta aristolochiae*, *Byasa alcinous*, and *Battus philenor*), 5 species from the genus *Papilio*, 1 species from the genus *Graphium* (family Papilionidae), and 2 species from other Lepidoptera families. Multiple alignments for each orthogroup were created using MAFFT (v7.505)^34^ and removed difficult alignment regions using trimAl (v1.4. rev15).^35^ The single-copy genes were extracted from each orthogroup, and the multiple alignment results were concatenated to estimate the maximum likelihood phylogenetic tree using IQ-TREE (v2.2.5).^36^ The target orthologs were 5,396 groups, and the model was Q.plant + F + R9 (automatic selection by IQ-TREE) with 1,000 bootstrap numbers. Additionally, the phylogenetic tree was converted to a timescale using r8 s (v1.81).^37^ Fossil information and branching ages from previous studies were used as calibration points. Specifically, the divergence between *Byasa alcinous* and *Iphiclides podalirius* was set at 56 Mya estimated by TimeTree.^38^ Finally, Café v5^39^ was used to analyze expansion/contraction in gene family size based on phylogenetic trees and orthogroup combinations. To investigate changes in gene family size related to aristolochic acid utilization, we searched for orthogroups that showed significant expansion or contraction in the clade containing the 3 aristolochic acid-utilizing species.

### 2.5. Positive selection analysis

Positively selected genes (PSGs) were identified from single-copy genes predicted by Sonicparanoid (v2.0.4).^33^ We first aligned the amino acid sequences of 5,396 single-copy genes of 11 lepidopteran species (Table S3) using ClustalOmega (v1.2.4)^40^ and the DNA sequences using PAL2NAL (v14).^41^ The ratio (ω) of nonsynonymous (dN) to synonymous (dS) substitutions was estimated using the codeml program in the PAML (v4.10.7)^42^ to detect positively selected genes in the clade containing *Pachliopta aristolochiae*, *Byasa alcinous*, and *Battus philenor*. A species phylogenetic tree based on single-copy orthologous genes was generated, and the corresponding node (node 20) representing the 3 aristolochic acid-utilizing species was labelled as the foreground branch. Codon alignments were analyzed with codeml under 2 models: the branch-site model (model = 2, NSsites = 2, fix_omega = 0), which allows positive selection on specific sites along the foreground branch, and the corresponding null model (model = 2, NSsites = 2, fix_omega = 1, omega = 1), which assumes neutral evolution. For each gene, codeml was run under both models, and log-likelihood values (lnL) were extracted from the output files. Likelihood ratio tests (LRTs) were conducted by calculating twice the difference in log-likelihoods (2Δℓ = 2 × [lnL_alt − lnL_null]), and statistical significance was assessed using a chi-square distribution with one degree of freedom. Genes were considered positively selected if the LRT statistic exceeded 3.841 (*P* < 0.05). Furthermore, GO analysis was performed using clusterProfiler (v4.10.0)^43^ for 417 PSGs using *Drosophila* database org.Dm.eg.db (v3.18.4).

### 2.6. Transcriptomic sequencing and expression analysis

The entire hindwing was sampled for RNA extraction on wandering and pupal stages, and the midgut on fifth instar larvae. RNA extraction was performed using TRI reagent (Sigma) in the same manner as Nishikawa *et al*.^19^ and Iijima *et al*.^23^ The extracted and DNase I (TaKaRa, Japan) treated RNA was sent to Macrogen Japan Corporation for library preparation by TruSeq stranded mRNA (paired-end, 101 bp) and sequenced by Illumina platform. The obtained RNA-seq reads were quality-checked by FastQC (v0.12.1),^44^ mapped by STAR (v2.7.10b),^45^ and the expressions of genes were quantified using RSEM (v1.3.1).^46^ These RNA-seq data were also used for gene annotation (see above; Table S2). In addition, to search for genes involved in the formation of the red spot, we sampled 3 individuals each from the red and black regions of the hindwings on pupal day 14 (P14) (Figure S12), performed RNA-seq, and obtained expression levels using the same method described above. We used DESeq2 (v3.14)^47^ to examine differentially expressed genes (DEGs) whose expression was greater in the red spot.

### 2.7. Identification of red pigments in *P*. *aristolochiae*

Red areas of the hindwings of *P*. *aristolochiae* were isolated from 10 individuals and heated in a 70% methanol solution at 50, 60, 70, and 80 °C to extract the pigments, which were then purified and used as analysis samples. High-performance liquid chromatography (HPLC) system (Prominence, Shimadzu, Kyoto) was employed for the elution of red pigments, with chromatograms at each absorption wavelength detected using a photodiode array detector (Prominence SPD-M20A, Shimadzu). Subsequently, precise mass analysis was conducted using high-performance liquid chromatography-electrospray ionization mass spectrometry (HPLC-ESI-MS) (Ultimate 3000, Dionex).

### 2.8. Functional analysis by RNAi using in vivo electroporation

siDirect (http://sidirect2.rnai.jp/) was used to design the siRNAs for each gene. The designed siRNA was synthesized by FASMAC Co., Ltd. (Kanagawa, Japan). The RNA powder received was dissolved in Nuclease-Free Water (Thermo Fisher, Ambion), adjusted to 500 μM, and stored at −20 °C. The sequence information of the siRNA used is listed in Table S11. A glass capillary (Narishige, GD-1 Model, Glass Capillary with Filament) was processed into a needle shape by heating it at HEATER LEVEL 66.6 using a puller (Narishige, PP-830 Model). The capillary was filled with siRNA, which was adjusted to 250 μM. About 4 μl of siRNA was injected into the left hindwing under a stereomicroscope using a microinjector (FemtoJet, eppendorf). Then, siRNA was introduced into only the positive pole side of the electrode by applying voltage (5 square pulses of 7.5 V, 280 ms width) using an electroporator (Cellproduce, electrical pulse generator CureGine). A PBS gel (20× PBS: NaCl 80 g, Na2HPO4 11 g, KCl 2 g, K2HPO4 2 g, DDW 500 ml; 1% agarose) was placed on the dorsal side of the hindwing and a drop of PBS was placed on the ventral side of the hindwing. The detailed method follows that described in the previous paper.^48^

## 3. Results

### 3.1. Genome assembly and gene annotation of *P. aristolochiae* and *B. alcinous*

Using GenomeScope 2.0^49^ to estimate genome size and heterozygosity, we found that *P. aristolochiae* has a genome size of 313.7 Mb and a heterozygosity rate of 0.59%, while *B. alcinous* has a genome size of 257.7 Mb and a heterozygosity rate of 0.88% (Figure S1). Next, de novo genome assembly produced a total genome size of 312.1 Mb with a scaffold N50 of 7.8 Mb for *P. aristolochiae* and a genome size of 257.6 Mb with a scaffold N50 of 9.5 Mb for *B. alcinous* (Fig. 1b; Table S3). Additionally, the completeness of the assemblies was evaluated using BUSCO (v5.4.3), with both species showing a 98.9% completeness score based on single-copy genes (Table S4).

Gene annotation identified an estimate 13,497 genes for *P. aristolochiae* and 14,669 in *B. alcinous*, with a gene annotation completeness score exceeding 98% based on BUSCO (v5.4.3) single-copy genes (Fig. 1b; Tables S5 and S6). Comparative genome analysis of the assembled genomes of *P. aristolochiae* and *B. alcinous* with the chromosome-level genome of the closely related species *P. xuthus* and *P*. *machaon* revealed that, although synteny is largely conserved, events of chromosome fusions and fissions were suggested.(Fig. 1c; Figure S2). Synteny also appears largely conserved between *P. aristolochiae* and *B. alcinous* (Figure S3), but further detailed analysis is limited by the current scaffolding level of these genomes.

### 3.2. Gene family expansion and contraction of *P. aristolochiae* and *B. alcinous*

**Fig. 2.**
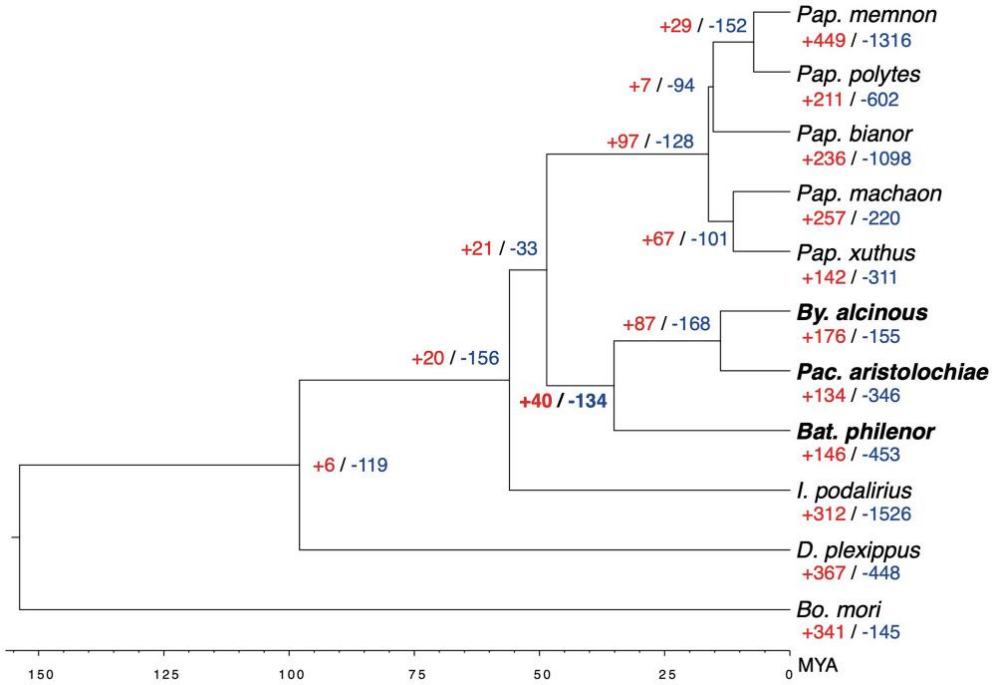
Summary of all expansion and contraction gene family across 11 lepidopteran species. Expansion and contraction numbers are shown in bold for nodes containing 3 toxic butterflies (node 20: the common ancestor of *Byasa alcinous*, *Pachliopta aristolochiae*, and *Battus philenor*). Red letters indicate the number of gene groups that are expanding, and blue letters indicate the number of gene groups that are contracting.

We conducted a comparative genomic analysis using whole-genome assemblies and annotations from nine species of the Papilionidae, along with *Danaus plexippus* and *Bombyx mori* data available from NCBI RefSeq (Table S3). First, we performed phylogenetic analysis and divergence time estimation based on concatenated single-copy genes, confirming that the phylogenetic relationships and divergence times of the Papilionidae tribes—Papilionini (including *Papilio memnon*), Troidini (including *Pachliopta aristolochiae* and *Byasa alcinous*), and Leptocircini (*Iphiclides podalirius*)—are consistent with previously reported studies (Figs. 1d and  2).^8^

Among the 11 lepidopteran species analyzed, only *P. aristolochiae*, *B. alcinous,* and *B. philenor* utilize aristolochic acid. One indicator of adaptive evolution is the increase or decrease in gene copy number.^50^ In the clade of *P. aristolochiae*, *B. alcinous,* and *B. philenor* (the Troidini clade), observed changes in gene copy numbers may be linked to the evolution of toxin utilization. Therefore, we identified ortholog groups across these 11 species and examined gene groups with increased or decreased gene numbers specifically in the Troidini clade. We identified a total of 12,990 ortholog groups, with 6,805 groups shared across all 11 species. Among them, 40 groups showed an increase in gene number (expanded gene groups: EGGs), and 134 showed a decrease in the Troidini clade compared to other clades, though no significant differences in overall gene number changes were observed between the Troidini clade and others (Fig. 2). Notably, 31 groups showed a statistically significant (*P* < 0.05) increase in gene count, while 112 groups showed a significant decrease (*P* < 0.05) in the Troidini clade (Table 1; Tables S7 and S8).

**Table 1.** Significantly expanded gene family in *Pachliopta aristolochiae*, *Byasa alcinous*, and *Battus philenor*, and the number of genes in 11 lepidopteran species.

Group_ID	GeneCount												*P*-value	GeneName(Bmor)
	Bm	Dp	Ip	Bp	Pa	Ba	Px	Pma	Pb	Pp	Pme	Total		
g185	3	1	0	**7**	**3**	**4**	1	5	0	1	1	26	9.87E-05	PiggyBac transposable element-derived protein 3 (LOC119629365)
g21	12	0	2	**9**	**15**	**11**	10	15	1	0	4	79	0.0001214	Uncharacterized LOC134199102 (LOC134199102)
g14	9	8	6	**14**	**13**	**11**	7	9	8	8	1	94	0.00028108	Uncharacterized LOC101744925 (LOC101744925)
g163	1	1	1	**4**	**6**	**7**	1	2	2	1	2	28	0.00048785	Olfactory receptor 56 (Or-56)
g178	1	2	1	**4**	**5**	**4**	2	2	2	2	2	27	0.00048785	Chemosensory protein 4 (CSP4)
g179	1	1	1	**5**	**4**	**6**	1	1	2	3	2	27	0.00048785	Synaptic vesicle 2-related protein (LOC101744459)
g63	2	4	3	**6**	**6**	**10**	2	2	5	3	1	44	0.00206284	Attacin 1 (Attacin1)
g82	1	1	4	**6**	**6**	**6**	2	4	3	4	2	39	0.00206284	Lysozyme (Lzm)
g16	2	5	4	**36**	**7**	**8**	4	2	15	4	6	93	0.00458531	Transmembrane protease serine 3-like (LOC105841499)
g1052	1	1	1	**2**	**1**	**3**	1	1	0	1	0	12	0.00702312	Dynein light chain roadblock-type 1 (LOC101736322)
g431	1	1	0	**5**	**1**	**3**	1	1	1	2	2	18	0.00702312	Olfactory receptor 6 (Or-6)
g4763	1	1	0	**3**	**3**	**1**	0	2	0	0	0	11	0.00702312	PiggyBac transposable element-derived protein 2-like (LOC134200054)
g539	2	1	1	**3**	**1**	**2**	1	1	2	1	1	16	0.00702312	Juvenile hormone esterase (LOC101743027)
g590	2	1	0	**3**	**2**	**1**	1	1	1	2	1	15	0.00702312	Odorant receptor 4 (LOC101739731)
g688	1	0	1	**2**	**3**	**2**	1	1	1	1	1	14	0.00702312	Protein yellow-like (LOC101737681)
g719	1	1	0	**2**	**2**	**2**	2	1	1	2	0	14	0.00702312	Pyruvate kinase (LOC101742086)
g17	9	5	5	**13**	**10**	**10**	9	11	8	7	1	88	0.00793088	Protein takeout (LOC101743962)
g19	19	4	8	**11**	**17**	**11**	8	3	1	3	2	87	0.00793088	Probable RNA-directed DNA polymerase from transposon BS (LOC101740242)
g186	3	1	2	**3**	**2**	**5**	3	1	2	2	2	26	0.0140464	Aldo-keto reductase AKR2E4-like (LOC101740161)
g196	1	2	2	**3**	**4**	**2**	2	2	2	4	2	26	0.0140464	Cocoonase (Coc)
g212	1	4	1	**3**	**2**	**4**	2	2	2	2	1	24	0.0140464	Gamma-interferon-inducible lysosomal thiol reductase (LOC101738266)
g220	2	2	2	**4**	**3**	**3**	2	2	1	2	1	24	0.0140464	Fatty-acyl reductase (Far)
g225	2	4	1	**4**	**3**	**2**	2	2	2	1	1	24	0.0140464	Integument esterase 2 (ie2)
g86	11	1	1	**3**	**3**	**6**	2	4	1	2	3	37	0.0140464	protein gooseberry-n Euro (LOC101741552)
g105	2	0	1	**7**	**4**	**3**	4	3	5	3	2	34	0.0210629	Putative membrane protein (LOC100861566)
g135	3	0	0	**4**	**6**	**5**	3	2	4	2	1	30	0.0210629	Ecdysone oxidase-like (LOC119629782)
g141	1	1	2	**4**	**4**	**4**	4	4	1	3	2	30	0.0210629	Peroxidase (LOC101740756)
g90	2	1	2	**4**	**8**	**5**	5	2	3	3	2	37	0.0210629	Uncharacterized LOC105841660 (LOC105841660)
g92	3	4	1	**10**	**4**	**3**	3	3	4	1	1	37	0.0210629	Chymotrypsin (LOC110386035)
g50	8	4	3	**5**	**10**	**11**	3	1	1	3	2	51	0.0280664	Facilitated trehalose transporter Tret1-2 homolog (LOC134199081)
g94	2	0	4	**5**	**5**	**5**	4	5	2	4	1	37	0.0280664	Cuticular protein RR-2 motif 134 (CPR134)

GeneCount indicates the number of genes in each family for each species. A value of 0 indicates that the gene was lost after divergence from the common ancestor. The values shown in bold indicate the numbers of genes in *Pachliopta aristolochiae*, *Byasa alcinous*, and *Battus philenor*, which utilize aristolochic acid.

EGGs included those encoding membrane proteins such as *transmembrane protease serine 3-like*, *putative membrane protein*, and *facilitated trehalose transporter Tret1-2 homolog*, as well as various enzymes. In addition, 31 EGGs were examined by RNA-seq for expression in the midgut, wing disc of fifth instar larvae, and pupal wing in *P*. *aristolochiae* and pupal wing in *B*. *alcinous* (Figures S4 and S5). Specifically, in the midgut—which may play a key role in aristolochic acid utilization (metabolism, accumulation, etc.)—high expression was observed for genes including *synaptic vesicle 2-related protein*, *attacin 1*, *lysozyme*, *transmembrane protease serine 3-like*, *protein yellow-like*, and *putative membrane protein* (Figure S4).

### 3.3. Identification of positive selection genes (PSGs) in *P. aristolochiae* and *B. alcinous*

**Fig. 3.**
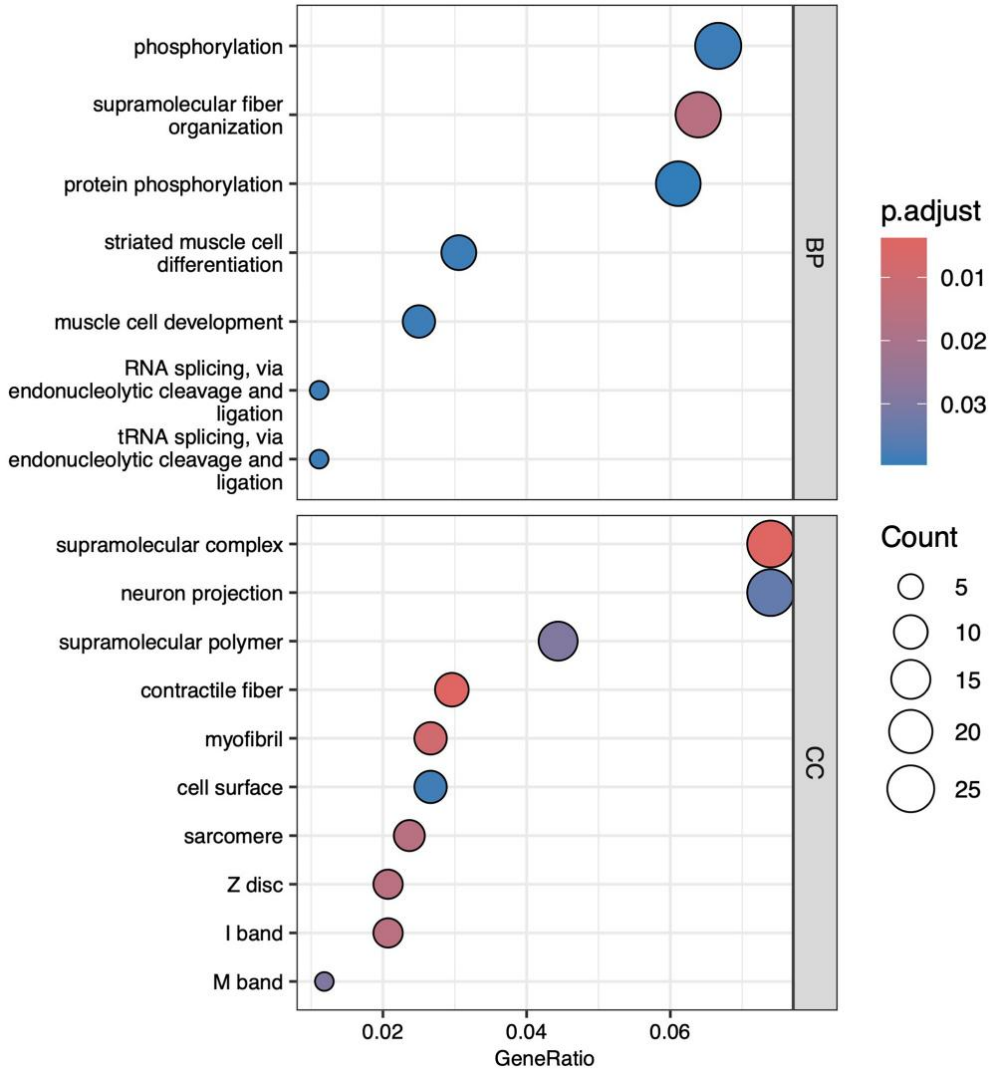
GO enrichment analysis of the positively selected 417 genes. Significantly enriched GO terms of biological process (BP) and cellular component (CC) are shown. No molecular function (MF) terms are displayed because none passed the significance threshold (adjusted *P*-value ≤ 0.05). Enrichment was performed using enrichGO with cut-off criteria of adjusted *P*-value (q-value) ≤ 0.05. The dotplot displays the top GO terms ranked by significance. GeneRatio indicates the proportion of input genes associated with each GO term. Count is the number of genes annotated to the GO term in the input set.

Next, among the 5,396 single-copy genes identified through ortholog estimation, we conducted a dN/dS analysis to search for genes under positive selection. As a result, 417 genes were inferred to be under positive selection in the Troidini clade (Table S9). GO analysis of the 417 PSGs revealed enrichment of Biological Process terms related to phosphorylation (GO:0016310) and protein phosphorylation (GO:0006468), which are key enzymatic processes involved in signal transduction and regulation of protein function. Additionally, enriched Cellular Component terms included supramolecular complexes (GO:0099080), which often comprise membrane-associated protein assemblies (Fig. 3). RNA-seq analysis was conducted to examine the expression of PSGs in the fifth instar larval midgut, larval wing discs, and pupal wings of *P. aristolochiae*, as well as in pupal wings of *B. alcinous* (Figures S6 and S7). In the larval midgut, which may be involved in the utilization of aristolochic acid (eg metabolism, accumulation), several PSGs exhibited midgut-specific high expression. These included *muscular protein 20, putative serine protease K12H4.7, probable peroxisomal acyl-coenzyme A oxidase 1, brachyuran, ankyrin repeat and MYND domain-containing protein 1 isoform X3, sodium/potassium/calcium exchanger 4, and a mitochondrial 2-oxoglutarate/malate carrier protein-like*, among others. In contrast, their expression was low in wing tissue (Figure S6).

### 3.4. Exploration and functional analysis of genes involved in wing warning colouration

**Fig. 4.**
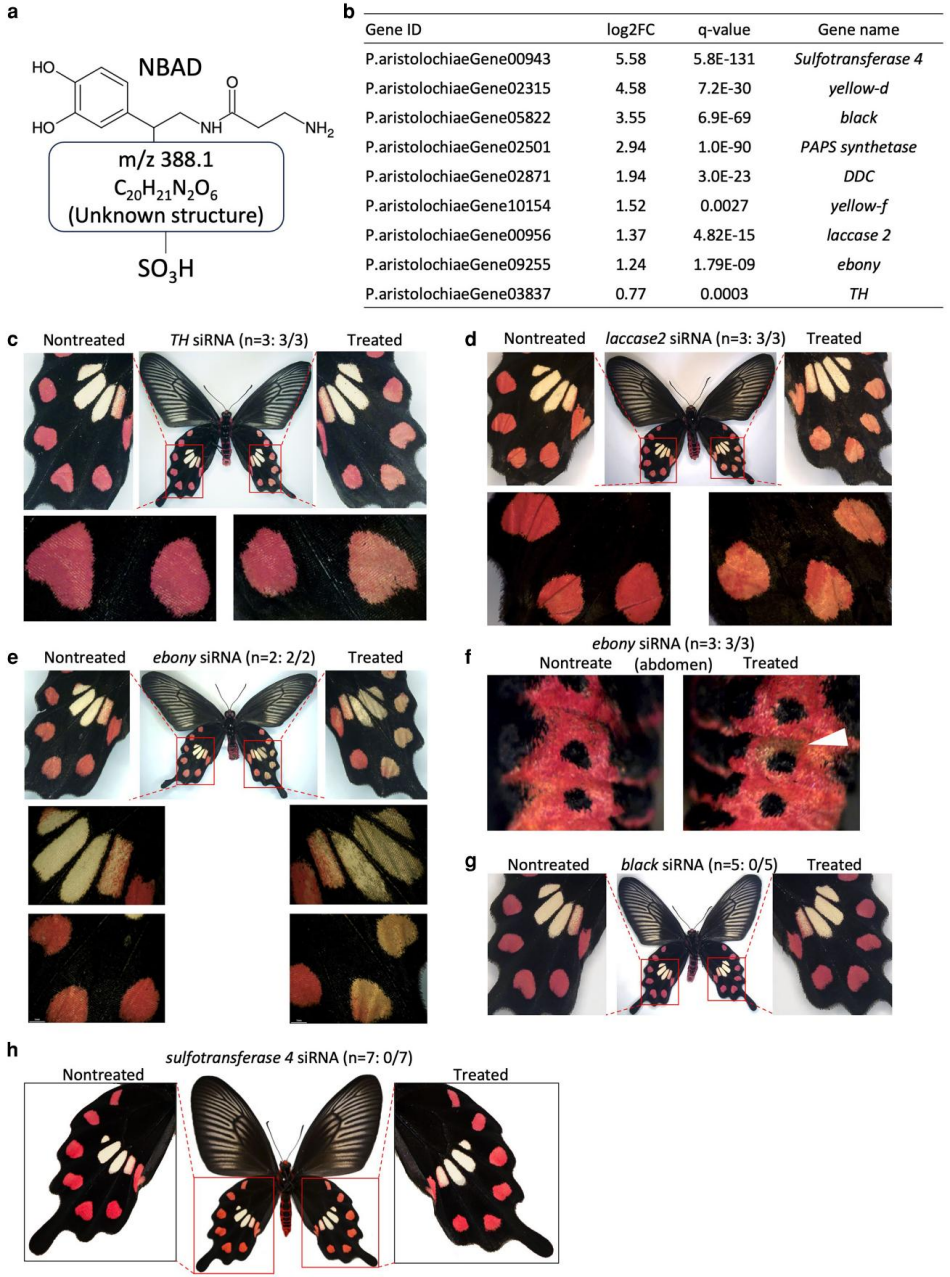
a) The predicted structure of pigments comprising the red spots in *Pachliopta aristolochiae*. b) Genes involved in NBAD synthesis and sulfate group addition that show higher expression levels in red spots compared to black regions. c–g) RNAi by electroporation targeting genes involved in NBAD synthesis. h) RNAi by electroporation targeting *sulfotransferase 4*.

The toxic butterflies *P. aristolochiae* and *B. alcinous* signal their toxicity to predators with warning colouration that accompanies the accumulation of aristolochic acid (Fig. 1a). To investigate the molecular mechanisms involved in the formation of the red spots in *P. aristolochiae* wings, an accurate mass analysis of the red pigment in the hindwing was conducted. This analysis revealed the presence of N-β-alanyldopamine (NBAD), an unknown substance (m/z 388.1), and a structure thought to contain sulfate groups (Fig. 4a; Figures S8–S11).

Next, RNA-seq analysis was performed on day 14 pupae (Figure S12) to identify DEGs between the red spots and black regions on the hindwings in *P. aristolochiae*. This analysis identified 442 genes that were highly expressed in the red spots (adjusted *P*-values < 0.01; Table S10). Among them were genes involved in NBAD synthesis, such as *yellow*, *black*, *DDC*, *laccase 2*, *ebony*, and *TH*, as well as *Sulfotransferase 4* and *PAPS synthetase*, which are associated with sulfate group addition (Fig. 4b).

To examine the functions of these genes, RNA interference (RNAi) experiments were conducted by introducing siRNA through electroporation on day 0 of pupation.^48^ Knockdown of *TH* and *laccase2* resulted in a noticeable fading of the red color in the red spots (Figs. 4c and 4d; Figures S13 and S16). Knockdown of *ebony* caused the pale-yellow spots to turn darker and the red spots to fade to a pinkish colour (Fig. 4e; Figure S14). Additionally, when *ebony* siRNA was introduced into red regions of the abdomen, the treated areas changed to brown (Fig. 4f; Figure S17). In contrast, no distinct phenotypic changes were observed with the introduction of *black* siRNA (Fig. 4g; Figure S15). These results indicate that NBAD plays a significant role in red pigment synthesis. Finally, knockdown of the sulfate-adding gene *Sulfotransferase 4* did not produce noticeable phenotypic changes in the red spots (Fig. 4h; Figure S18). However, in the current RNAi experiments, we did not measure the reduction of target gene expression. Therefore, caution is needed when interpreting the lack of observable phenotypic changes for genes such as *black* or *Sulfotransferase 4*.

### 3.5. Common genes in expanded gene groups (EGGs), positive selection genes (PSGs), and highly expressed genes in red spots (HEGs) in *P. aristolochiae*

Finally, we confirmed whether there were common genes among EGGs, PSGs, and HEGs by matching the gene IDs of *P. aristolochiae*. First, there were no common gene IDs between EGGs and PSGs. Next, when comparing EGGs and HEGs, we found 10 common gene IDs (Table S12). Among these, genes such as *cocoonase*, *glucose dehydrogenase*, and *facilitated trehalose transporter* were identified. Finally, there were 29 common gene IDs between PSGs and HEGs (Table S13).

## 4. Discussion

In this study, we performed whole-genome sequencing of the toxic butterflies *P*. *aristolochiae* and *B*. *alcinous* that utilize aristolochic acid. The genome sizes were comparable to those of other Papilionidae butterflies, measuring approximately 250 to 400 Mb (Fig. 1b; Table S3). While we did not construct the genome at the chromosomal level in this study, comparisons with closely related species that have their genomes assembled at the chromosomal level, such as *P*. *xuthus* and *P*. *machaon*, suggested the possibility of chromosomal fusion (Fig. 1c; Figure S2). Rearrangements such as chromosomal fusions have frequently been reported in Lepidoptera.^51,52^ Constructing chromosomal-level genomes for *P*. *aristolochiae* and *B*. *alcinous* and their close relatives in the future will allow for more detailed analyses.

How did the utilization of aristolochic acid, specifically the accumulation of toxins and warning colouration as a predator avoidance strategy, evolve in the family Papilionidae? In this study, we successfully identified candidate genes for the adaptive evolution of toxin utilization and warning colouration through 3 types of analyses: expanded gene families (EGGs), positively selected genes (PSGs), and highly expressed genes in red spots (HEGs). In addition to the previously available genome of *B*. *philenor*, we sequenced the genomes of *P*. *aristolochiae* and *B*. *alcinous* and incorporated them into our analyses. By analyzing 3 species that utilize aristolochic acid and 8 species that do not, we were able to perform branch-site tests with the aristolochic acid–utilizing clade designated as the foreground branch, as well as tests for clade-specific gene family expansions. This approach enabled us to distinguish species-specific changes from shared signals associated with aristolochic acid utilization.

By confirming the functions of EGGs and PSGs, we may identify genes associated with the utilization of aristolochic acid. To achieve this, experiments such as feeding knockout lines with diets containing aristolochic acid and diets without it will be necessary. Similar experiments have been conducted with *Pieris* species, where genes involved in detoxification have been identified.^53^ However, there is currently no information on detoxification or toxin utilization genes in the groups of *P*. *aristolochiae* and *B*. *alcinous*, leaving us without any clues regarding the causal genes. Therefore, we hope to gain insights into causal genes from the gene list we provided.

Additionally, HEGs include *distal-less* (P.aristolochiaeGene11051). *Distal-less* is known to be involved in the formation of eye spots in the butterfly *Bicyclus anynana*.^54^ Furthermore, several genes related to the Toll signalling pathway are listed among the HEGs (*easter*: P.aristolochiaeGene06357 and P.aristolochiaeGene07940; *Toll-7*: P.aristolochiaeGene11985; *Toll*: P.aristolochiaeGene04538). The Toll signalling pathway has been reported to be involved in melanin synthesis in silkworm larvae,^55^ suggesting it may play a role in the formation of warning colouration in the wings of *P*. *aristolochiae*. For the genes involved in spot formation on the wings, functional analysis can be conducted through RNAi using electroporation, similar to the knockdown of NBAD-related genes performed in this study (Fig. 4c–h). On the other hand, functional analyses of genes related to toxin utilization have not yet been conducted in the family Papilionidae, making it challenging to analyze the functions of numerous genes. Therefore, one idea is to use *Drosophila melanogaster* to analyze the functions of genes involved in toxin utilization and narrow it down to a few key genes before conducting functional analyses in the toxic butterflies of the Papilionidae family.

In this study, we found that the red pigment in the red spots of *P. aristolochiae* contains a substance that includes NBAD, and we were able to confirm the involvement of genes related to NBAD synthesis (ie *TH*, *laccase2*, and *ebony*) through functional analysis via RNAi. However, the structure of the red pigment beyond NBAD remains unclear (Fig. 4a). Analysis by HPLC–ESI–MS revealed 2 major peaks: one (peak 2) corresponding to NBAD, while the other (peak 1) remains unidentified (Figures S8–S11). The unknown portion of the chemical composition is C_20_H_21_N_2_O_6_, indicating a relatively large organic molecule that is expected to possess multiple aromatic rings, cyclic structures, and various functional groups. For instance, it is possible that metabolic products of aristolochic acid are utilized in this part, but determining this requires accurately predicting the chemical structure.

This study explored the genes involved in the utilization of aristolochic acid through genome sequencing, annotation, and comparative genomic analysis of 2 species of toxic butterflies that utilize aristolochic acid. In addition, genes associated with the formation of warning colours could be investigated and functionally analyzed. This research serves as a starting point for studies on the utilization of toxins and the formation of warning colouration, providing a list of genes potentially related to these processes for the first time.

## Supplementary Material

dsaf038_Supplementary_Data

## Data Availability

The raw sequence data and genome assemblies were deposited in DDBJ/NCBI/EMBL (BioProject: PRJDB19144 and PRJDB19142).
