# Supplementary material for: Whole genome analysis of toxic Papilionidae butterflies utilizing aristolochic acid, Pachliopta aristolochiae, and Byasa alcinous
Source: DNA Res. 2025 Dec 22;33(1):dsaf038. doi: 10.1093/dnares/dsaf038 (PMC12784068; doi:10.1093/dnares/dsaf038)
Supplement: dsaf038_Supplementary_Data [file dsaf038_supplementary_data.zip › PaBa_sup_r1.pdf]

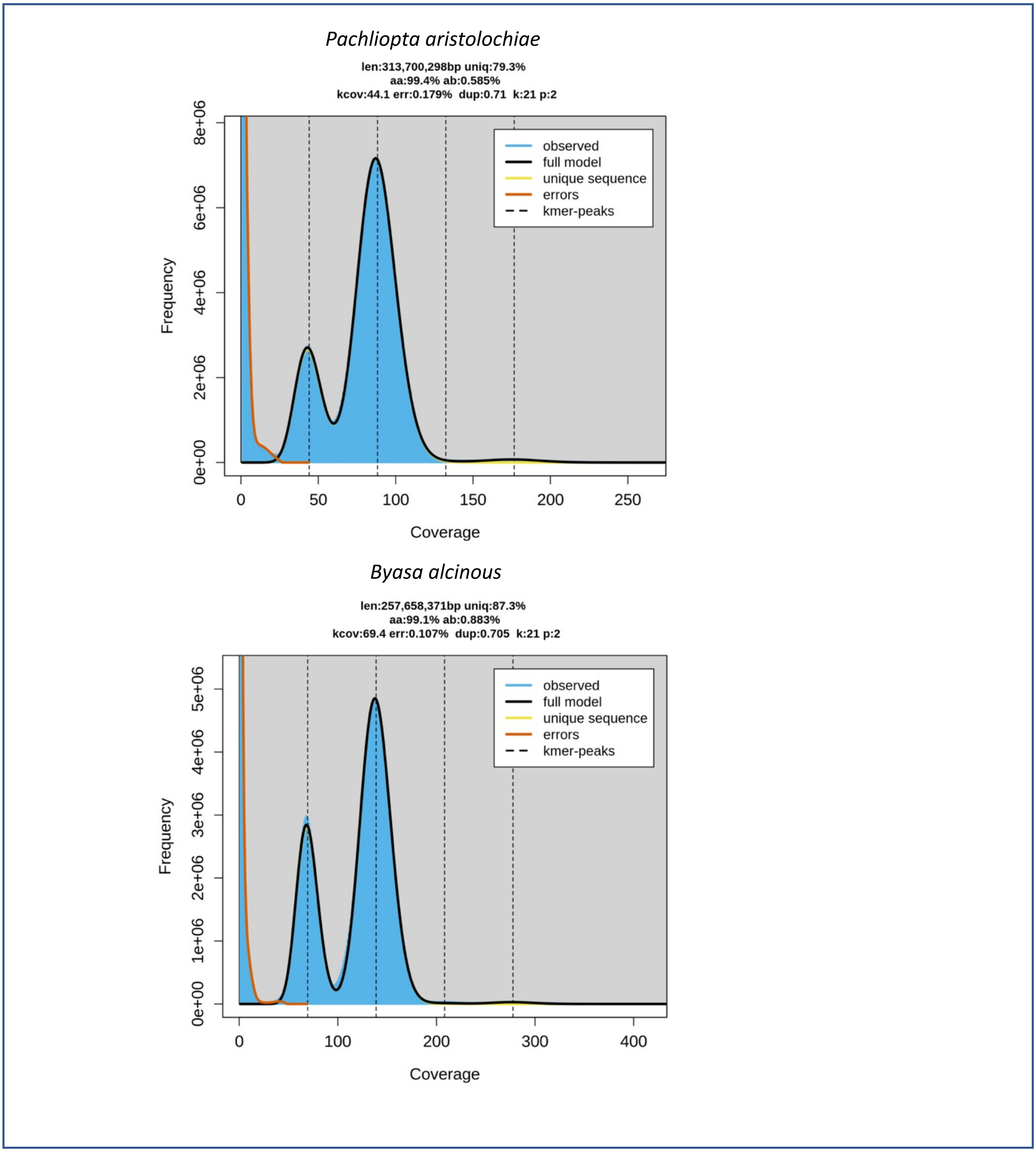

Supplementary Figure S1. Estimated genome size and heterozygosity of *Pachliopta aristolociae* and *Byasa alcinous* by GenomeScope. The 21-mer frequencies were calculated using Jellyfish v2.3.0 (Marçais and Kingsford 2011), and genome size was estimated using GenomeScope 2.0 (Ranallo-Benavidez et al. 2020). The estimated genome sizes were 314 and 258 Mbp in *P. aristolociae* and *B. alcinous*, respectively.

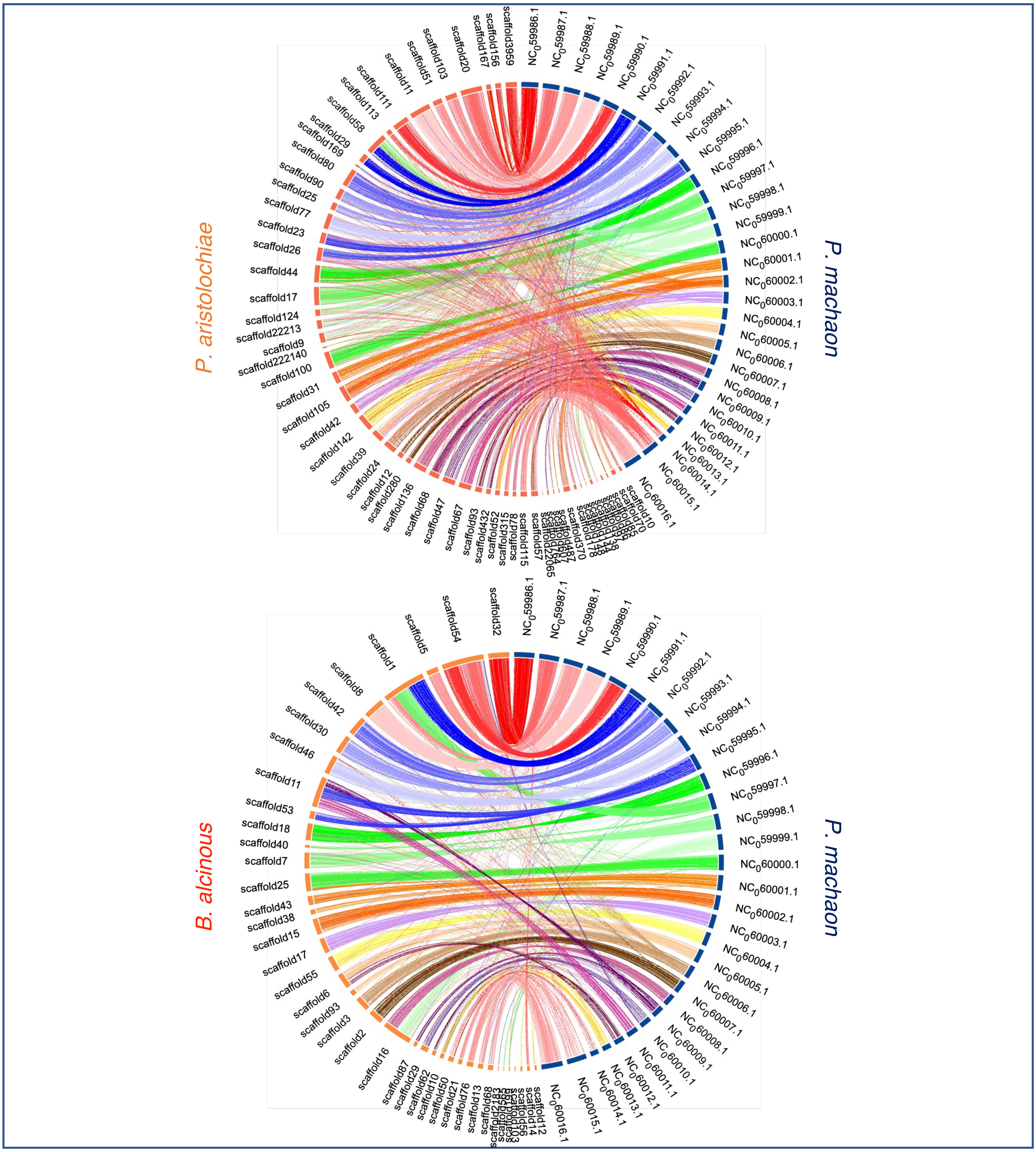

Supplementary Figure S2. Synteny plots between *P. aristolochiae* and *Papilio machaon*, and between *B. alcinous* and *P. machaon. P. machaon* genome is constructed at the chromosome level (NCBI RefSeq: GCF_912999745.1).

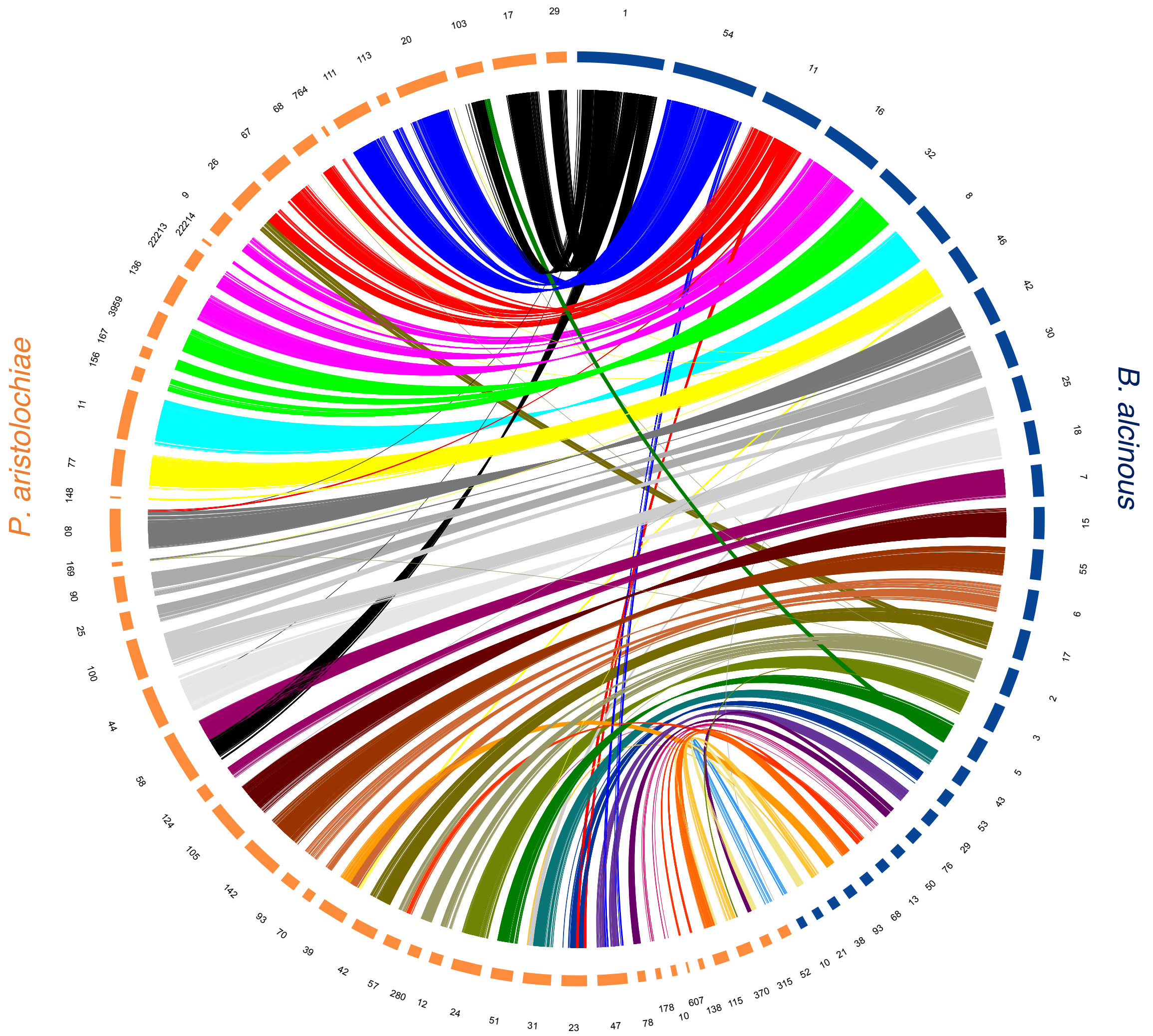

Supplementary Figure S3. Synteny plots between *P. aristolochiae* and *B. alcinous* constructed in this study.

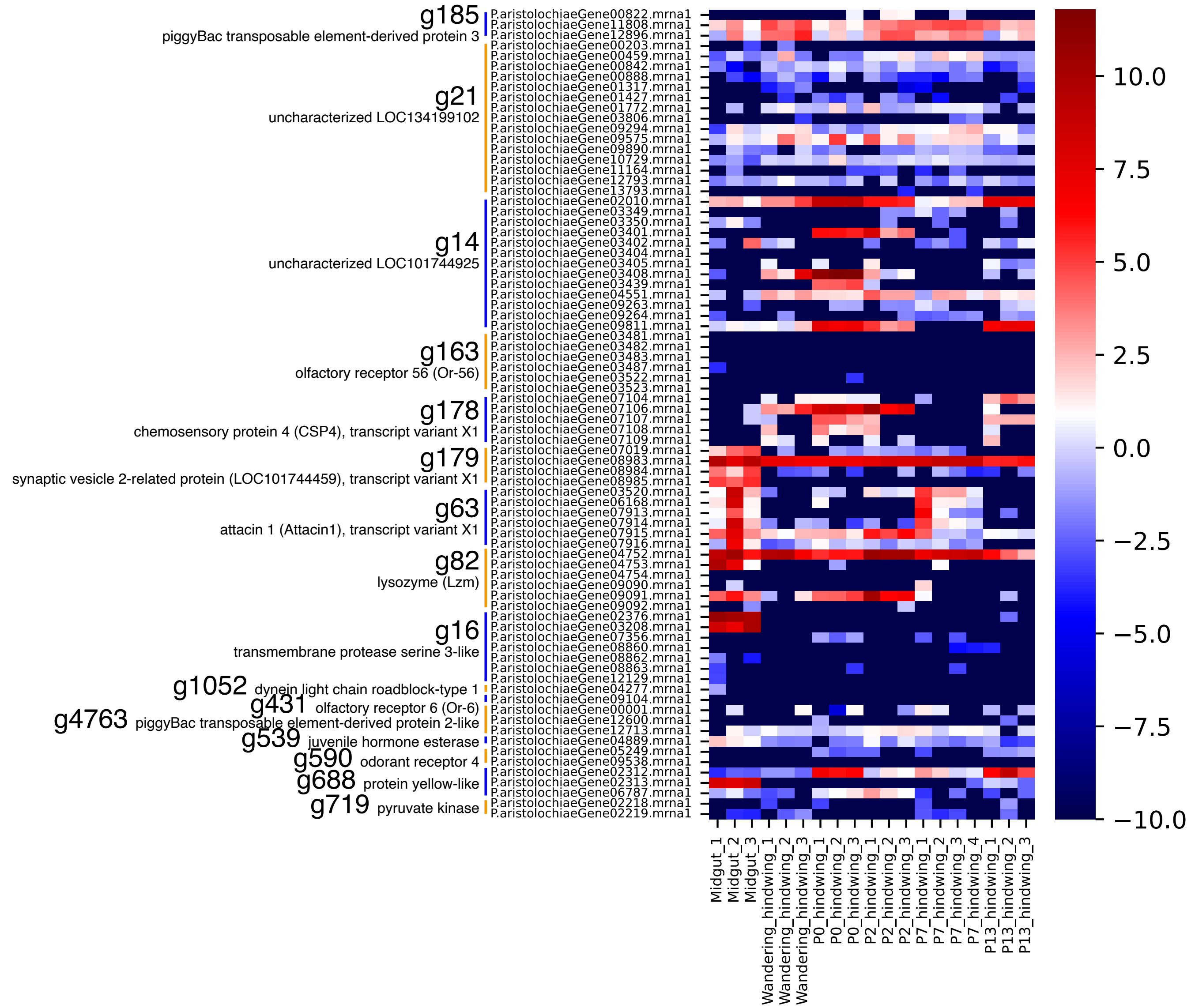

Supplementary Figure S4-1. Heatmap of gene expressions in expansion gene groups of *Pachliopta aristolociae* by RNA-seq. A heatmap showing the expression of three to four individuals at each stage: midgut of 5th instar larvae, hindwings of wandering stage, hindwings at pupal day 0 (P0), hindwings at pupal day 2 (P2), hindwings at pupal day 7 (P7), and hindwings at pupal day 13 (P13). The FPKM values are log2-transformed.

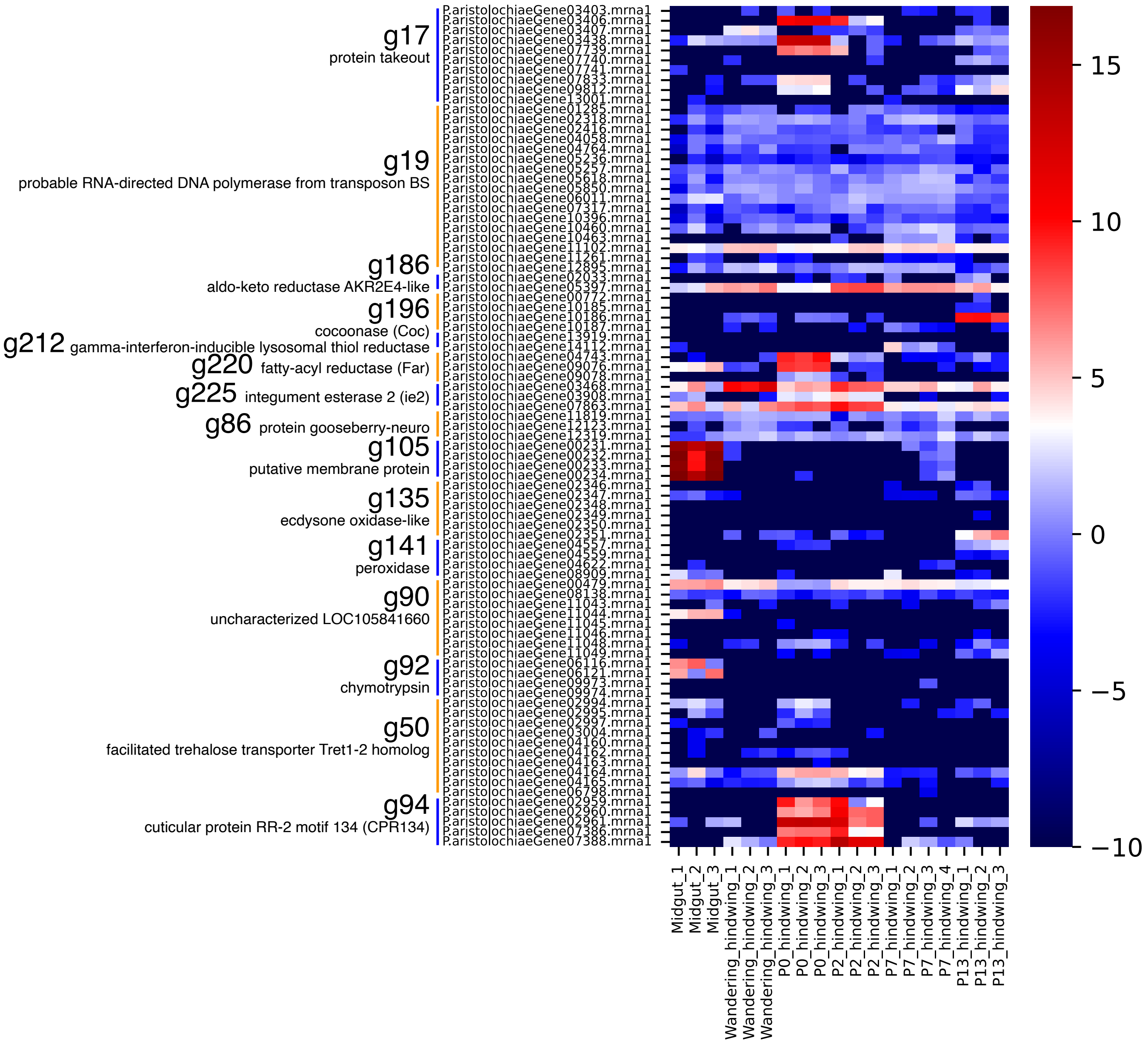

Supplementary Figure S4-2. Heatmap of gene expressions in expansion gene groups of *Pachliopta aristolociae* by RNA-seq. A heatmap showing the expression of three to four individuals at each stage: midgut of 5th instar larvae, hindwings of wandering stage, hindwings at pupal day 0 (P0), hindwings at pupal day 2 (P2), hindwings at pupal day 7 (P7), and hindwings at pupal day 13 (P13). The FPKM values are log2-transformed.

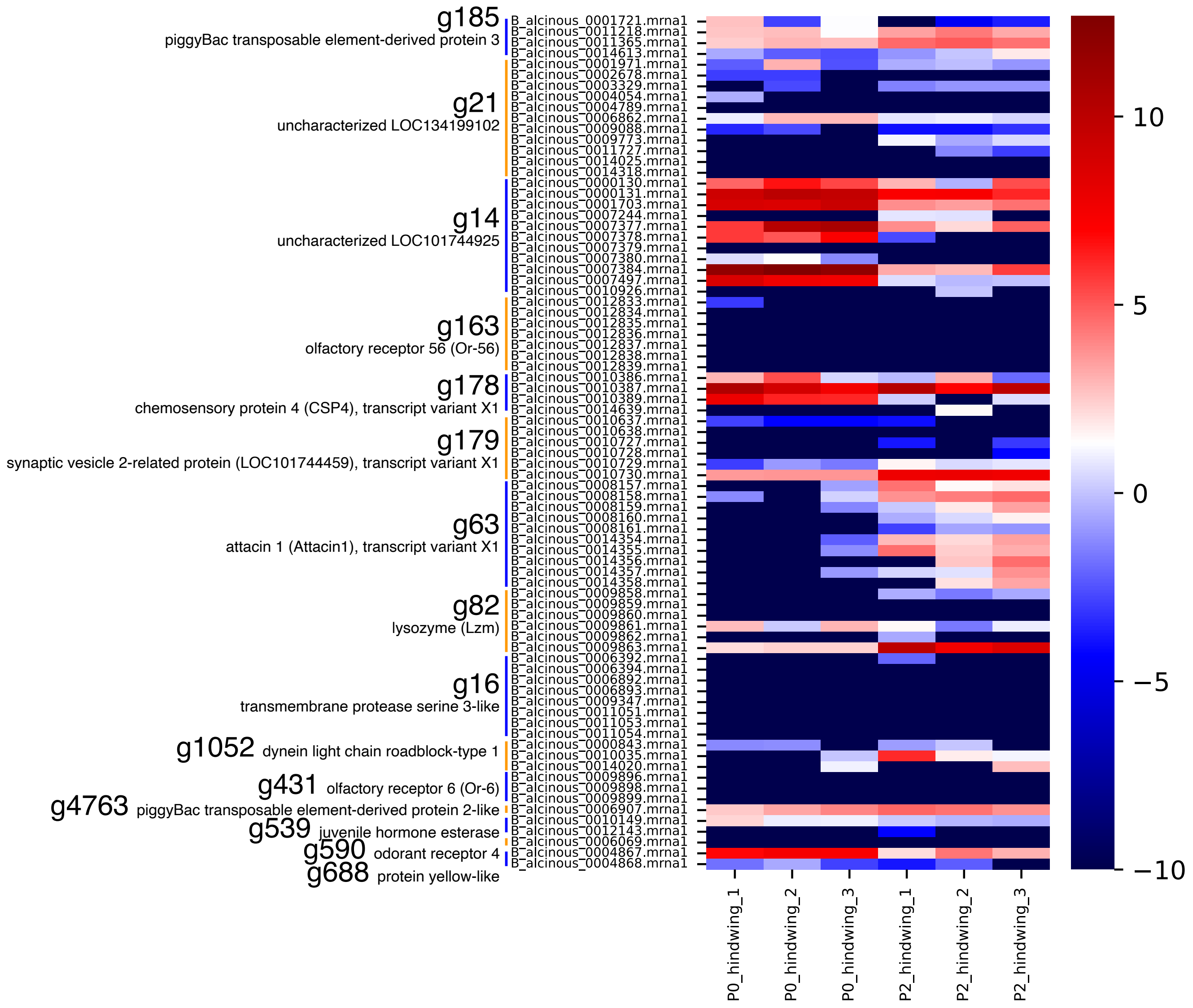

Supplementary Figure S5-1. Heatmap of gene expressions in expansion gene groups of *Byasa alcinous* by RNA-seq. A heatmap showing the expression of three individuals at each stage: hindwings at pupal day 0 (P0) and hindwings at pupal day 2 (P2). The FPKM values are log2-transformed.

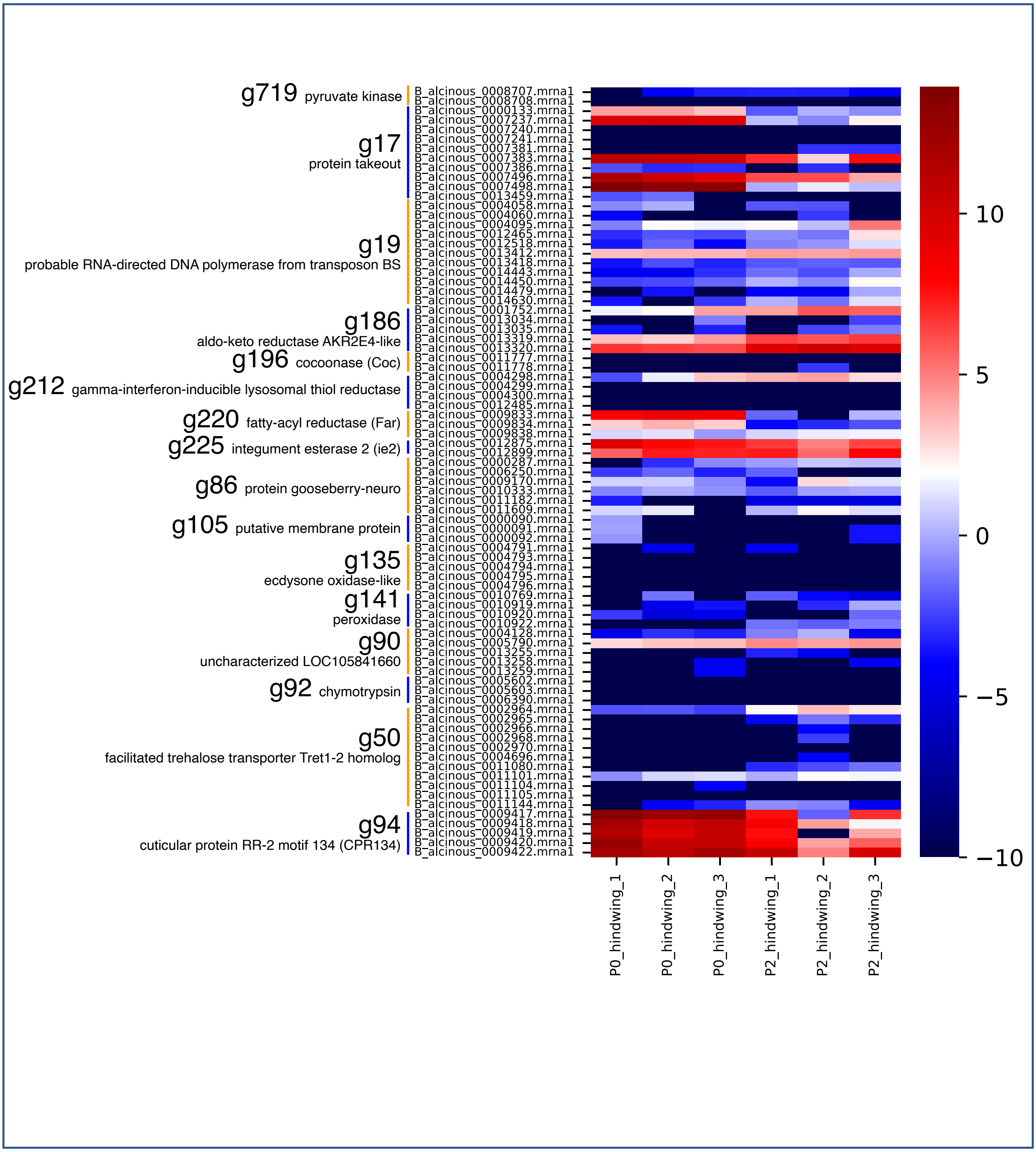

Supplementary Figure S5-2. Heatmap of gene expressions in expansion gene groups of *Byasa alcinous* by RNA-seq. A heatmap showing the expression of three individuals at each stage: hindwings at pupal day 0 (P0) and hindwings at pupal day 2 (P2). The FPKM values are log2-transformed.

Supplementary Figure S6

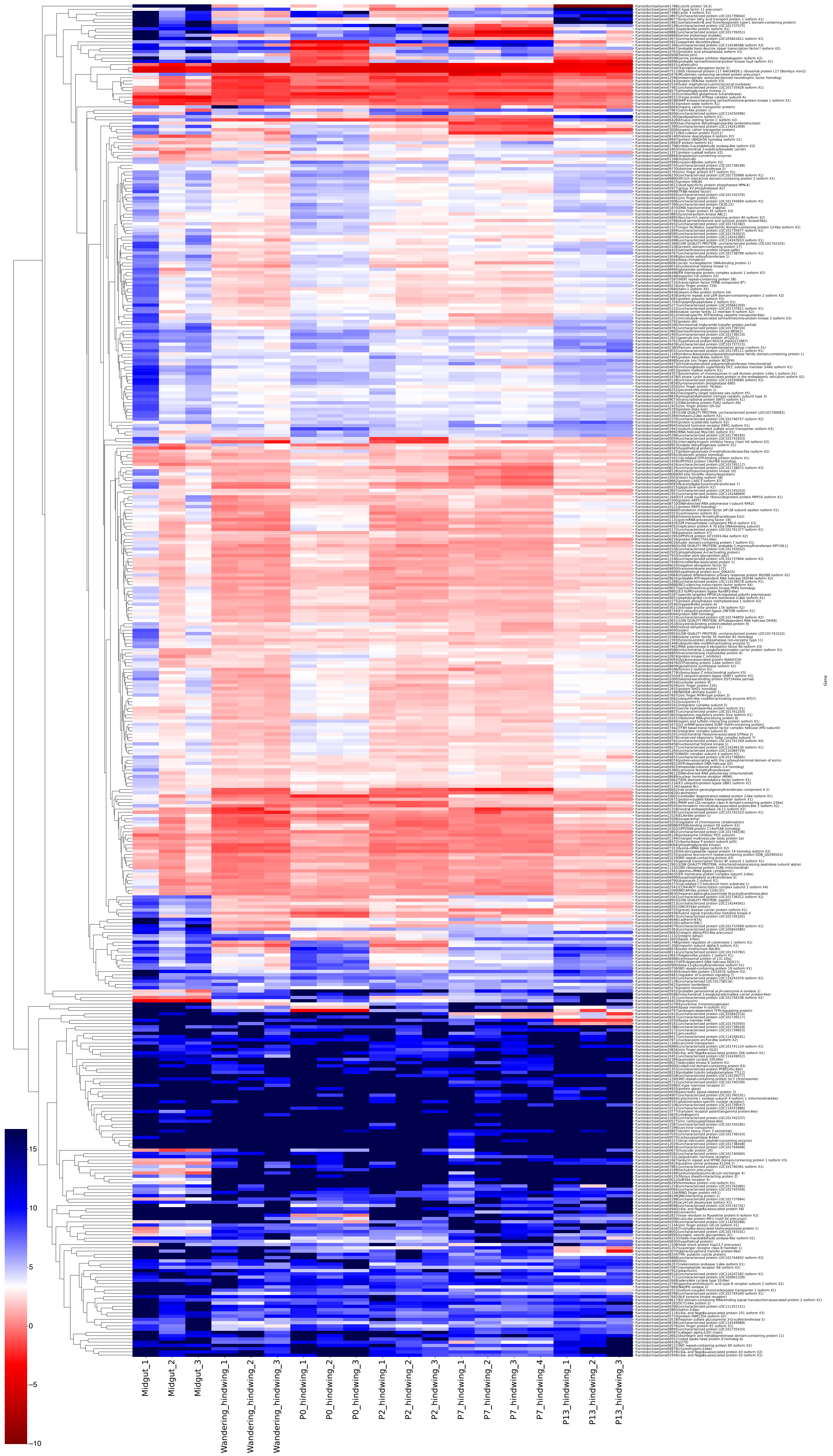

Supplementary Figure S6. Gene expression heatmap of positively selected genes in single-copy genes of *Pachliopta aristolociae*. A heatmap showing the expression of three to four individuals at each stage: midgut of 5th instar larvae, hindwings of wandering stage, hindwings at pupal day 0 (P0), hindwings at pupal day 2 (P2), hindwings at pupal day 7 (P7), and hindwings at pupal day 13 (P13). The FPKM values are log2-transformed.

Supplementary Figure S7

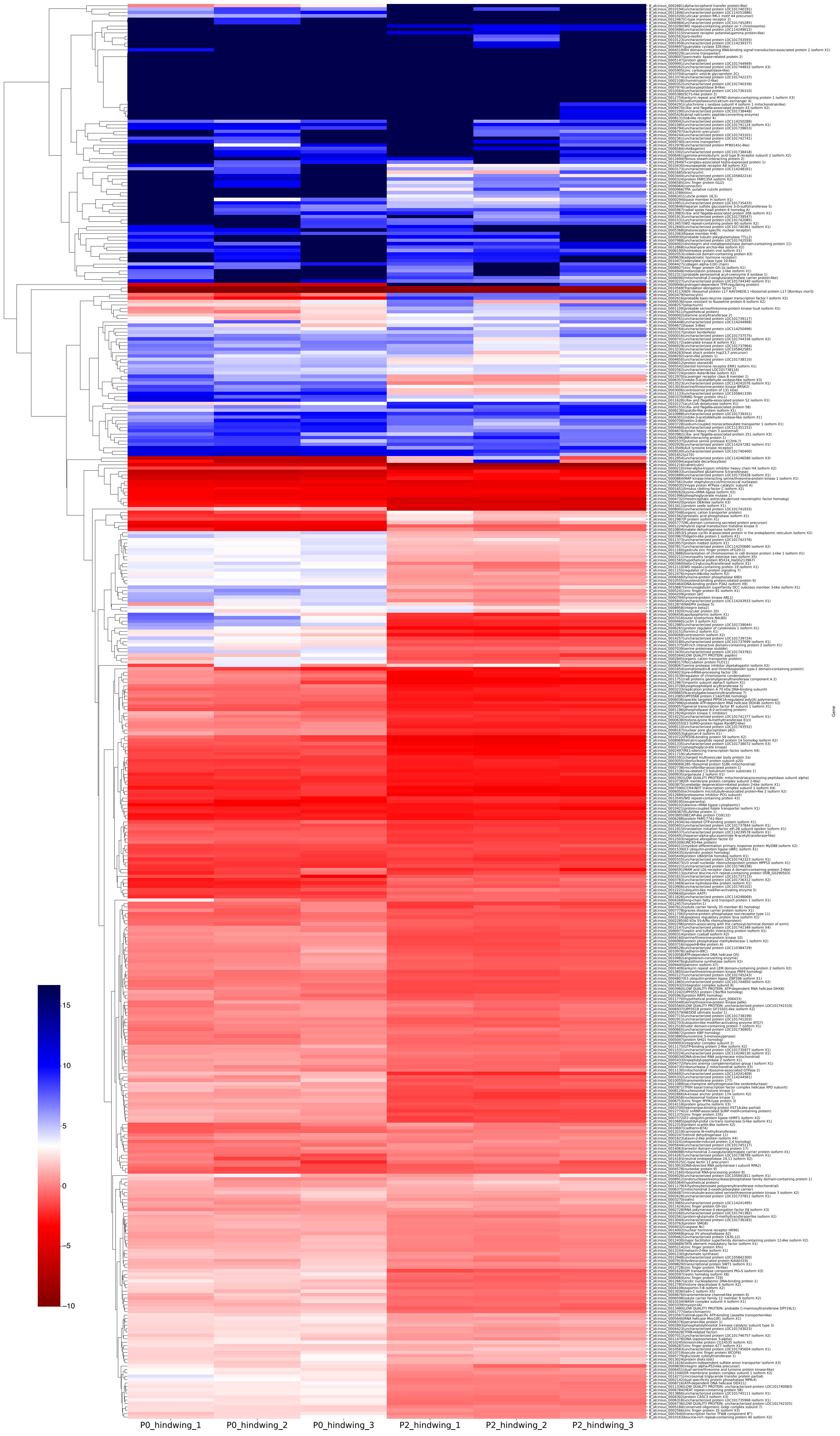

Supplementary Figure S7. Gene expression heatmap of positively selected genes in single-copy genes of *Byasa alcinous*. A heatmap showing the expression of three individuals at each stage: hindwings at pupal day 0 (P0) and hindwings at pupal day 2 (P2). The FPKM values are log2-transformed.

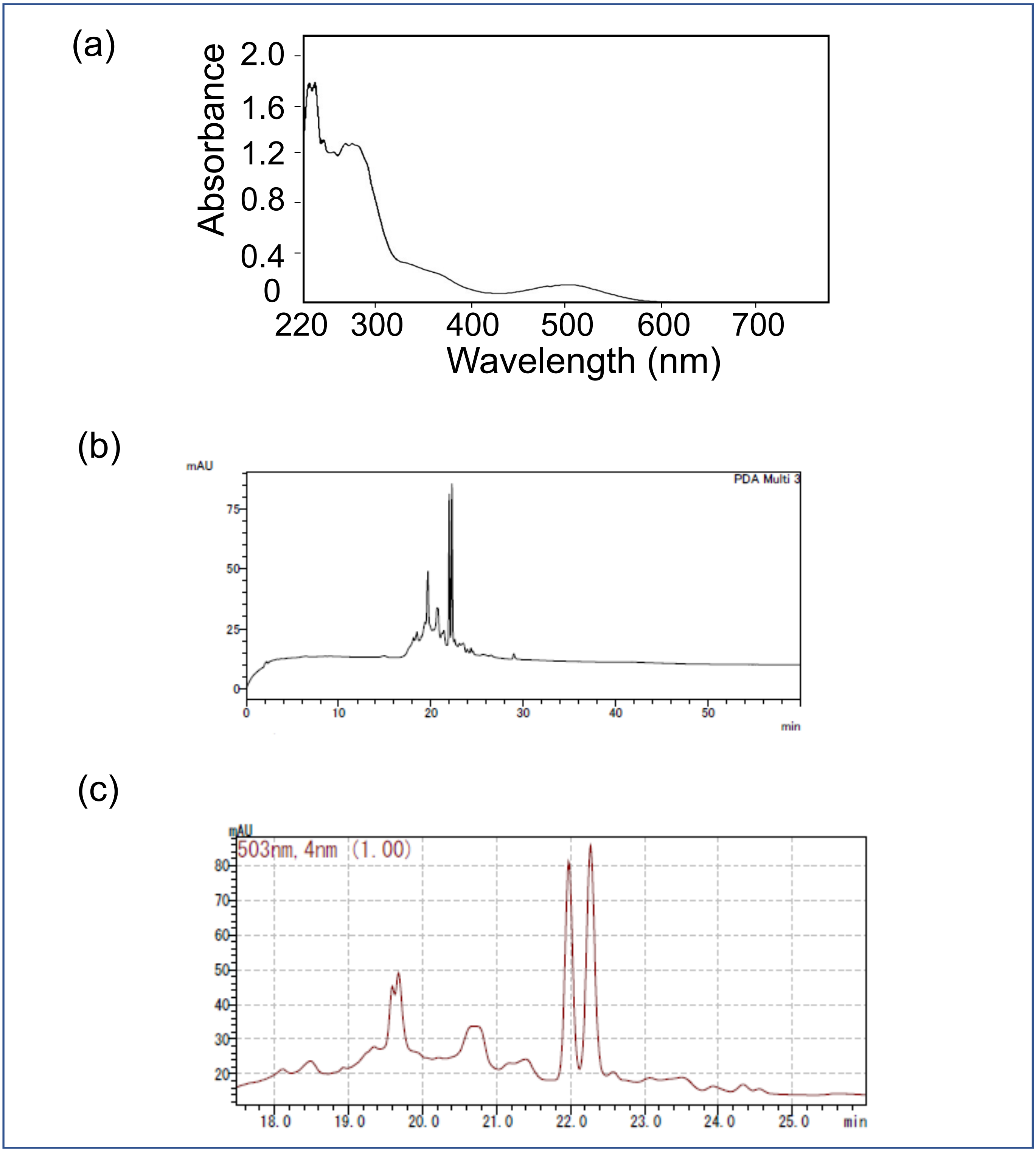

Supplementary Figure S8. Absorption wavelength of red pigment solution from *Pachliopta aristolociae* and separation of pigments by High Performance Liquid Chromatography (HPLC). (a) Absorption wavelength of red pigment extraction solution from *Pachliopta aristolociae*. A characteristic peak near 503nm is observed. (b) Separation by HPLC of red pigment. The chromatogram at 503nm is shown. The figure (c) enlarges the region around the retention time of 20 minutes, where mainly two peaks are observed.

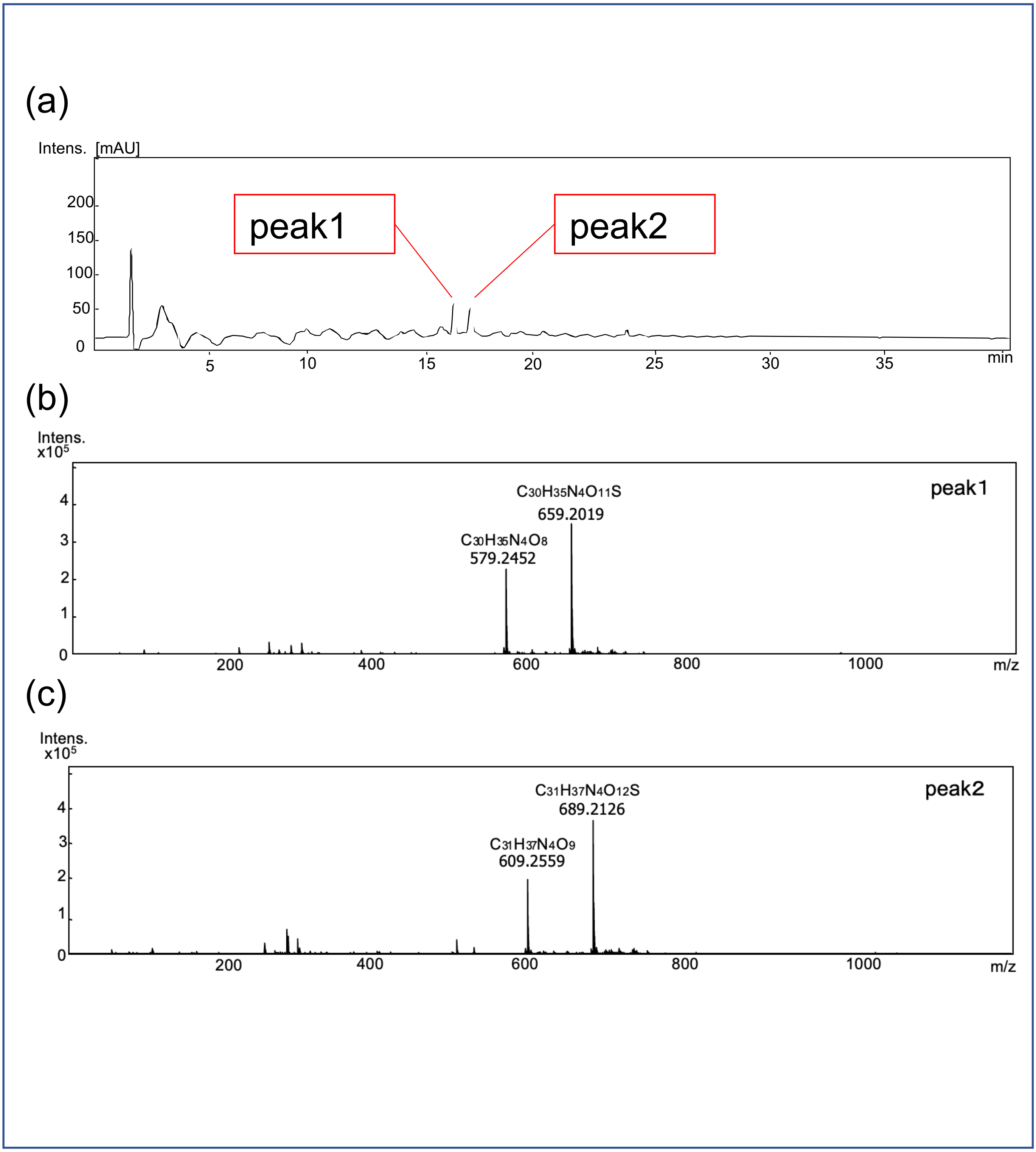

Supplementary Figure S9. Precise mass analysis of red pigment from *Pachliopta aristolociae* by HPLC-ESI-MS. (a) Chromatogram at 503nm in HPLC-ESI-MS. Mainly two peaks are observed. The precise mass and atomic composition are indicated for peak 1 (b) and peak 2 (c).

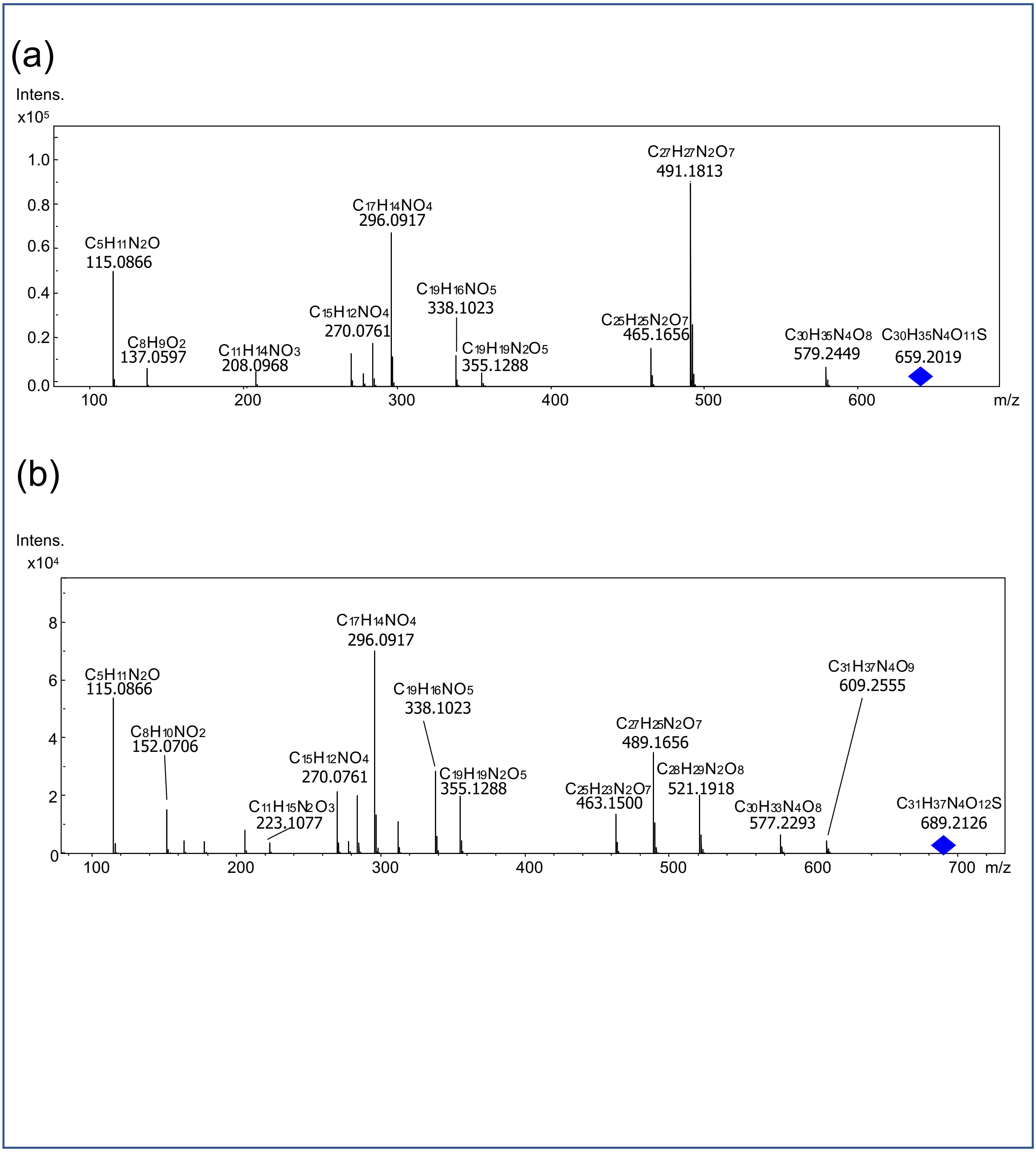

Supplementary Figure S10. MS/MS analysis of peak 1 and peak 2 of the red pigment from *Pachliopta aristolociae*. (a) Fragmentation pattern in MS/MS analysis of peak 1. (b) Fragmentation pattern in MS/MS analysis of peak 2. Peaks corresponding to the loss of SO3 were observed in both peaks. The blue diamond in the figure represents the precursor ion.

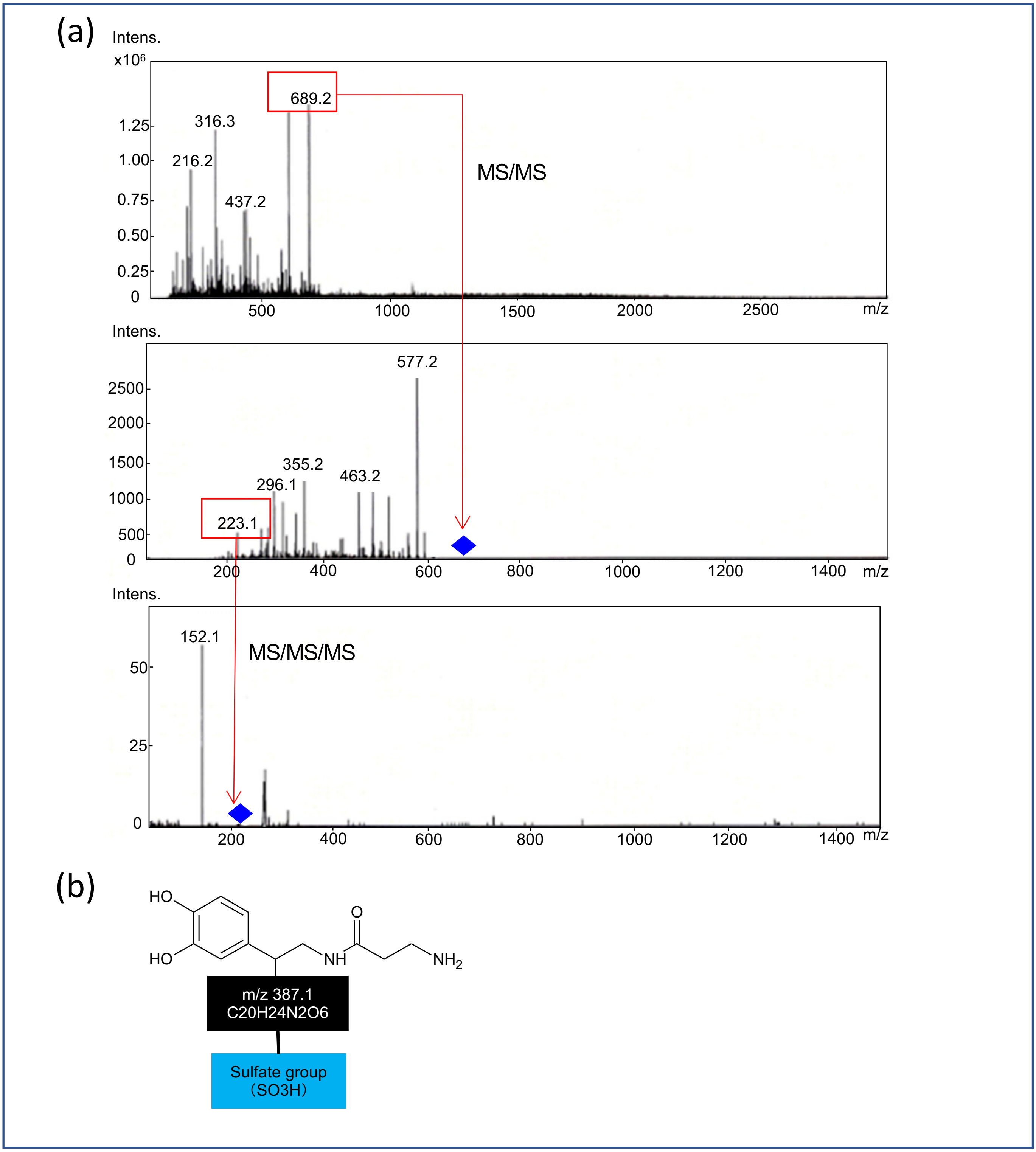

Supplementary Figure S11. MS/MS and MS/MS/MS analysis of peak 2. (a) Analysis of peak 2 by MS/MS revealed a fragment at m/z 223.1. Using this fragment as the precursor ion for MS/MS/MS analysis, a fragment at m/z 152.1 was obtained. The MS/MS/MS fragmentation pattern at m/z 223.1 matches the fragment pattern of NBAD. This suggests that NBAD is a component of the red pigment. Furthermore, based on the results from Figure S11 indicating the presence of a sulfate group, the structure of the red pigment can be predicted to be as shown in (b).

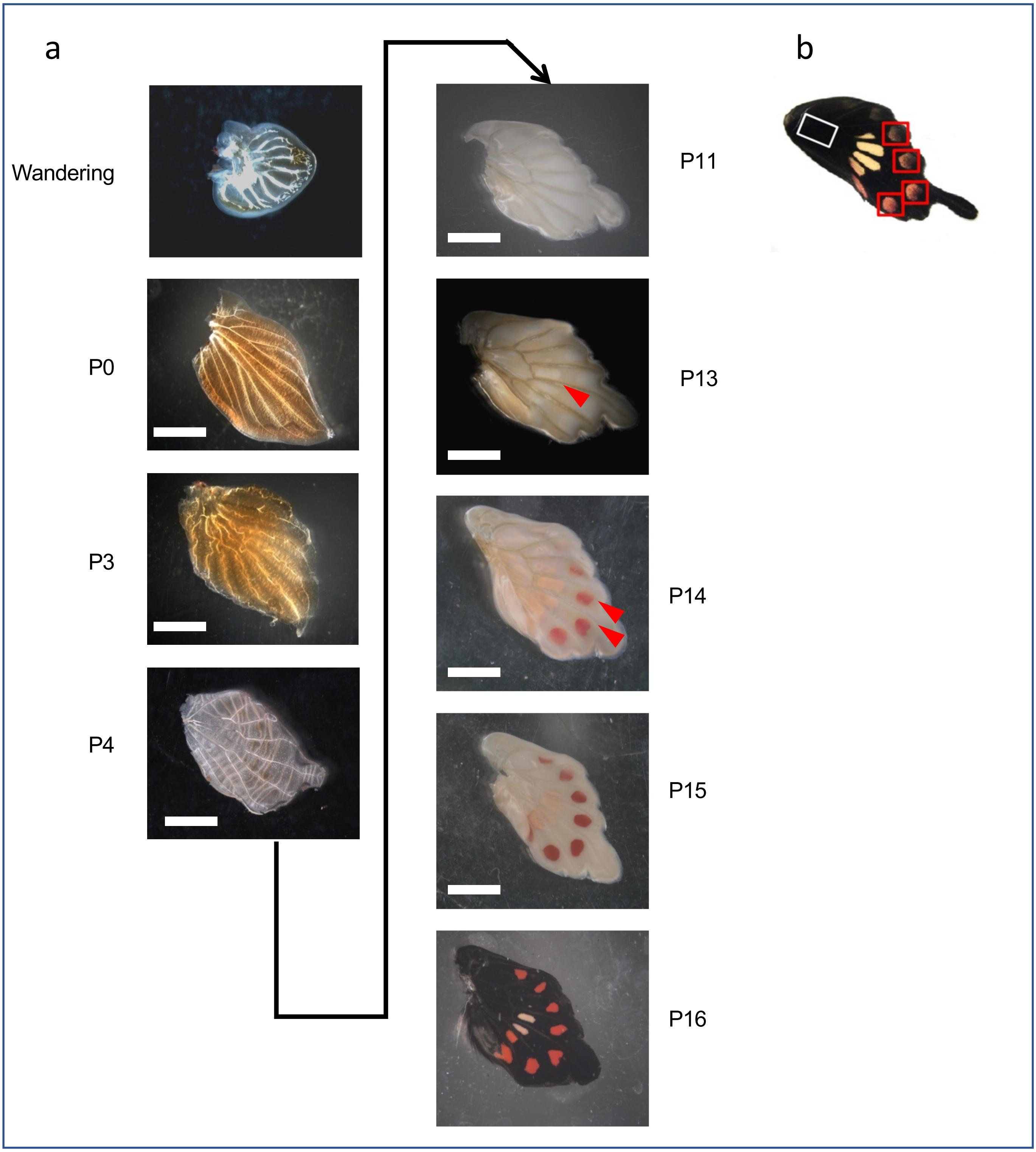

Supplementary Figure S12. (a) Hindwing development and coloration during the pupal stage in *Pachliopta aristolociae***.** P0 to P16 show the number of days after pupation. P15: Red spot coloration is complete. P16: Wing pattern coloration is completed as the black areas become colored. The scale bar represents 5 mm. (b) Wing region of adults corresponding to the region sampled in P14 to search for genes highly expressed in the red spots.

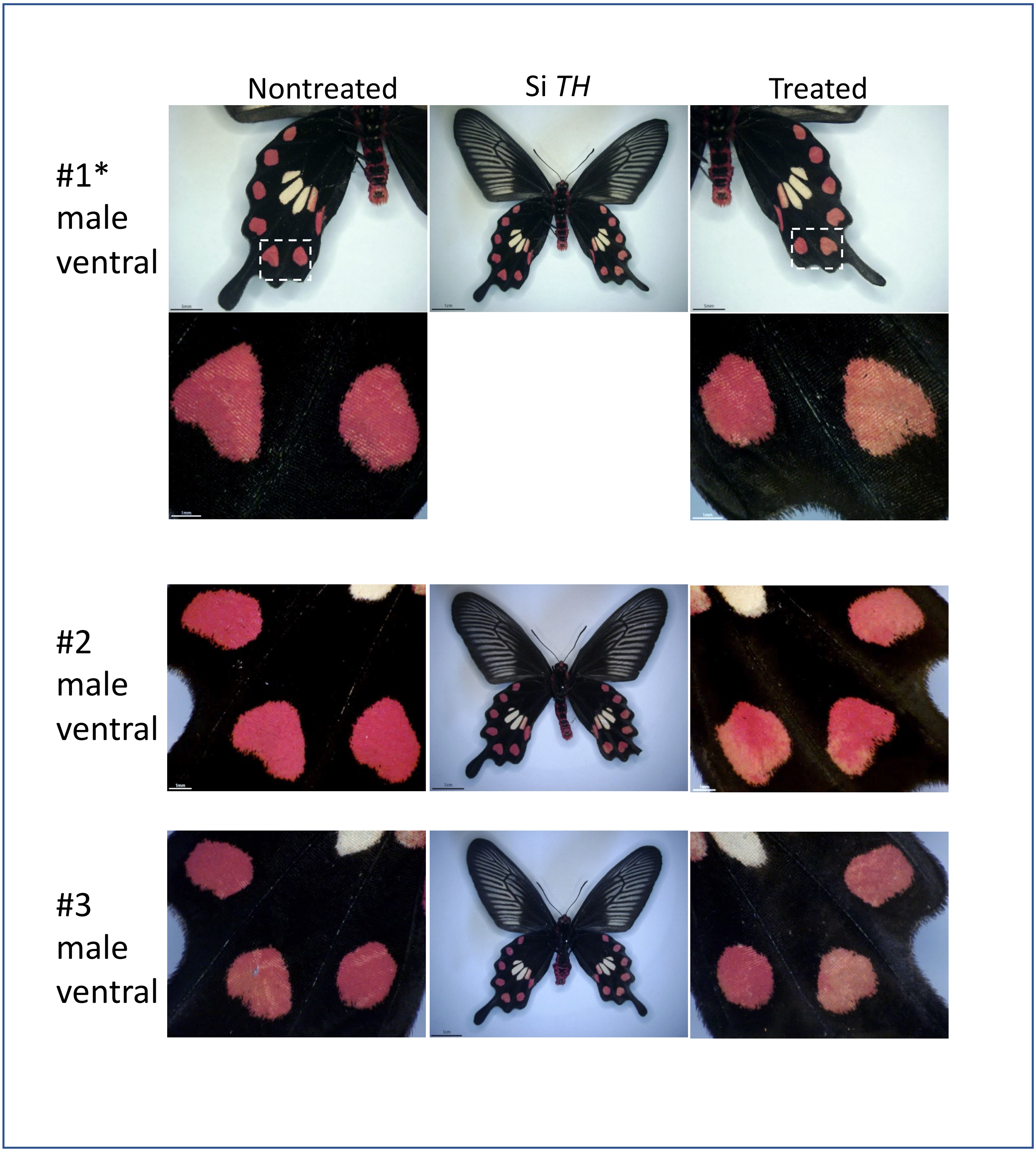

Supplementary Figure S13. Knockdown of *tyrosine hydroxylase* (*TH*) in the hindwings of *Pachliopta aristolochiae*. Other replicates of Fig. 4c. *#1 is the same individual as Fig. 4c.

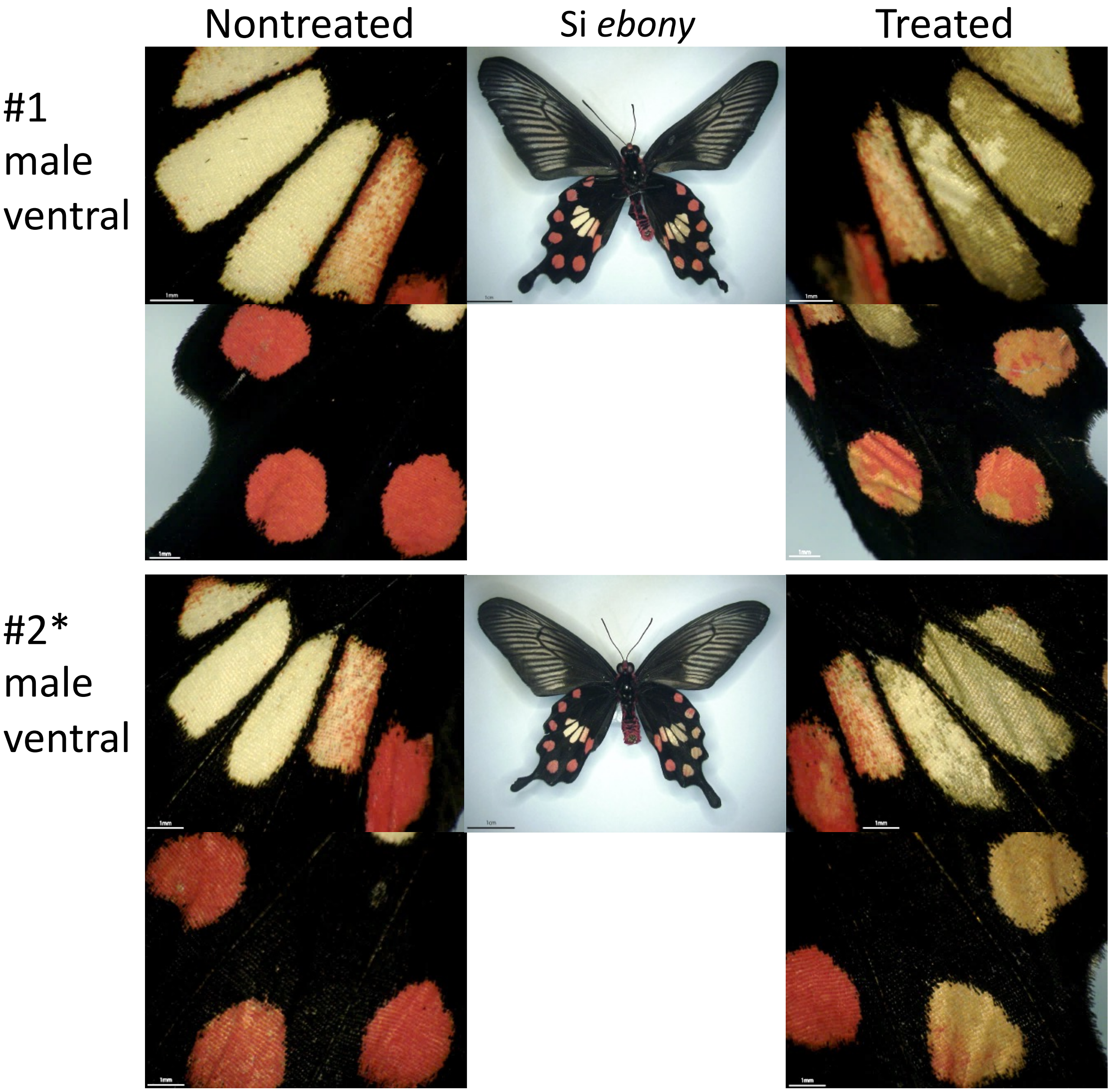

Supplementary Figure S14. Knockdown of *ebony* in the hindwings of *Pachliopta aristolochiae*. Other replicates of Fig. 4e. *#2 is the same individual as Fig. 4e.

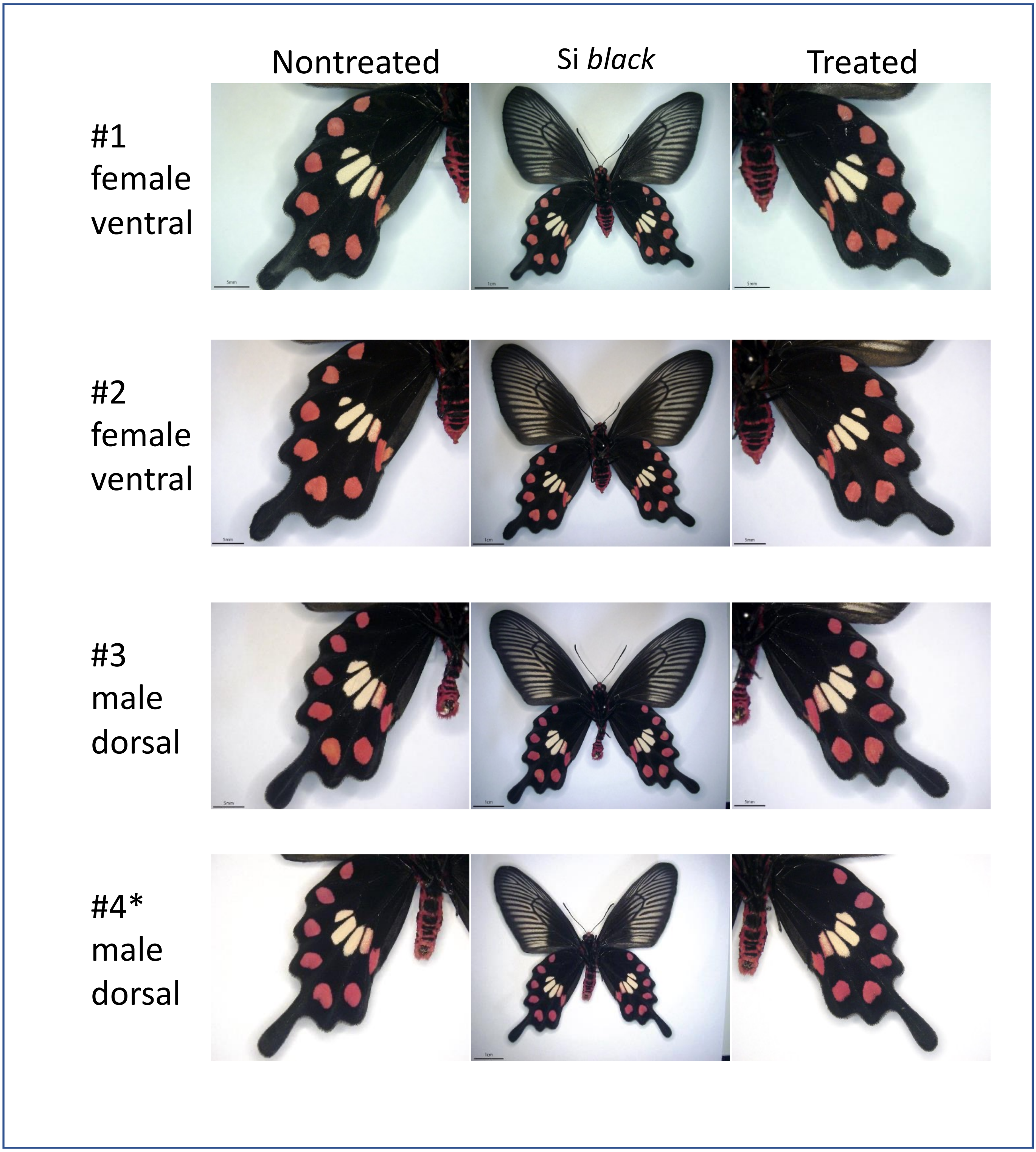

Supplementary Figure S15. Knockdown of *black* in the hindwings of *Pachliopta aristolochiae*. Other replicates of Fig. 4g. *#4 is the same individual as Fig. 4g.

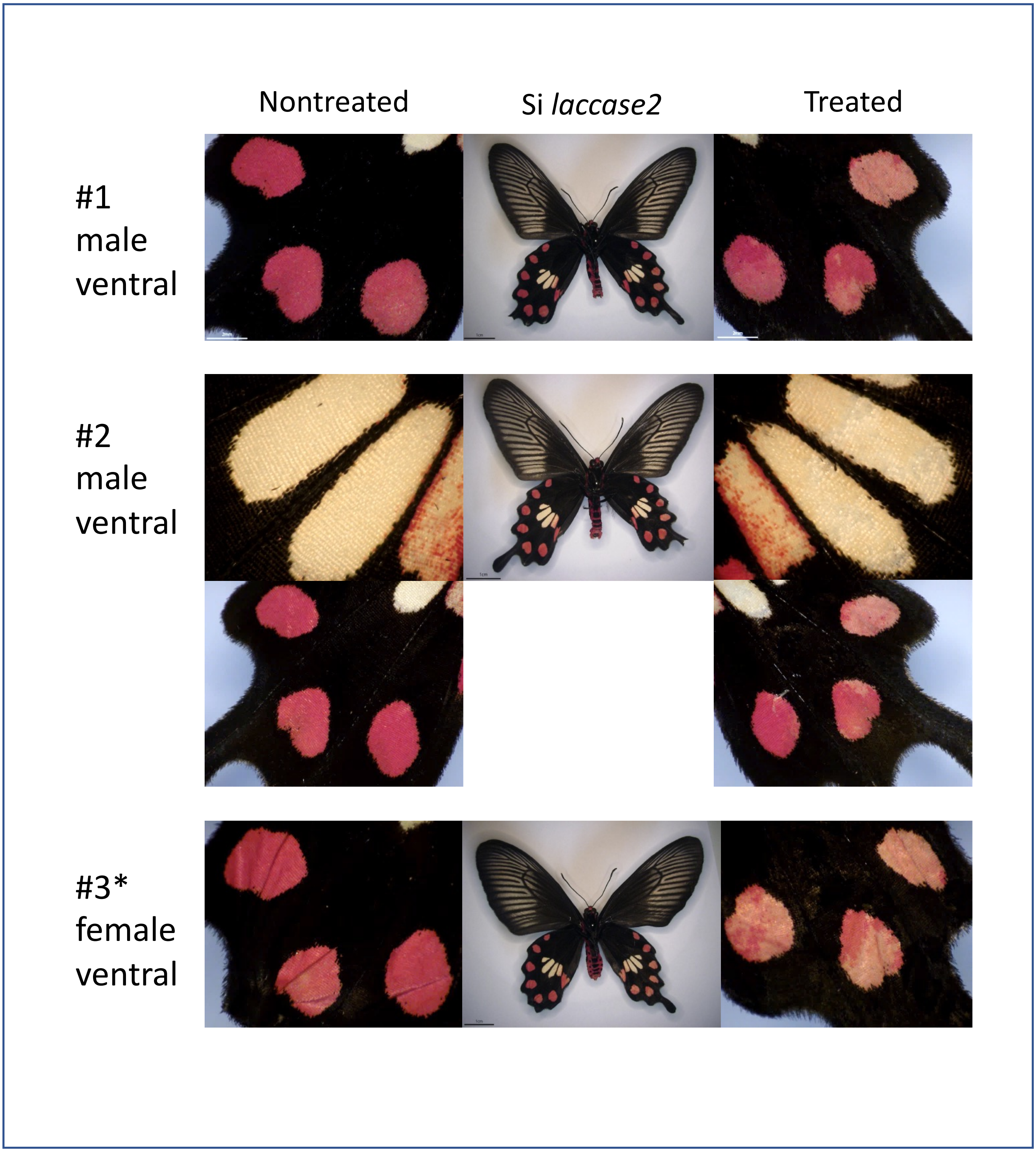

Supplementary Figure S16. Knockdown of *laccase2* in the hindwings of *Pachliopta aristolochiae*. Other replicates of Fig. 4d. *#3 is the same individual as Fig. 4d.

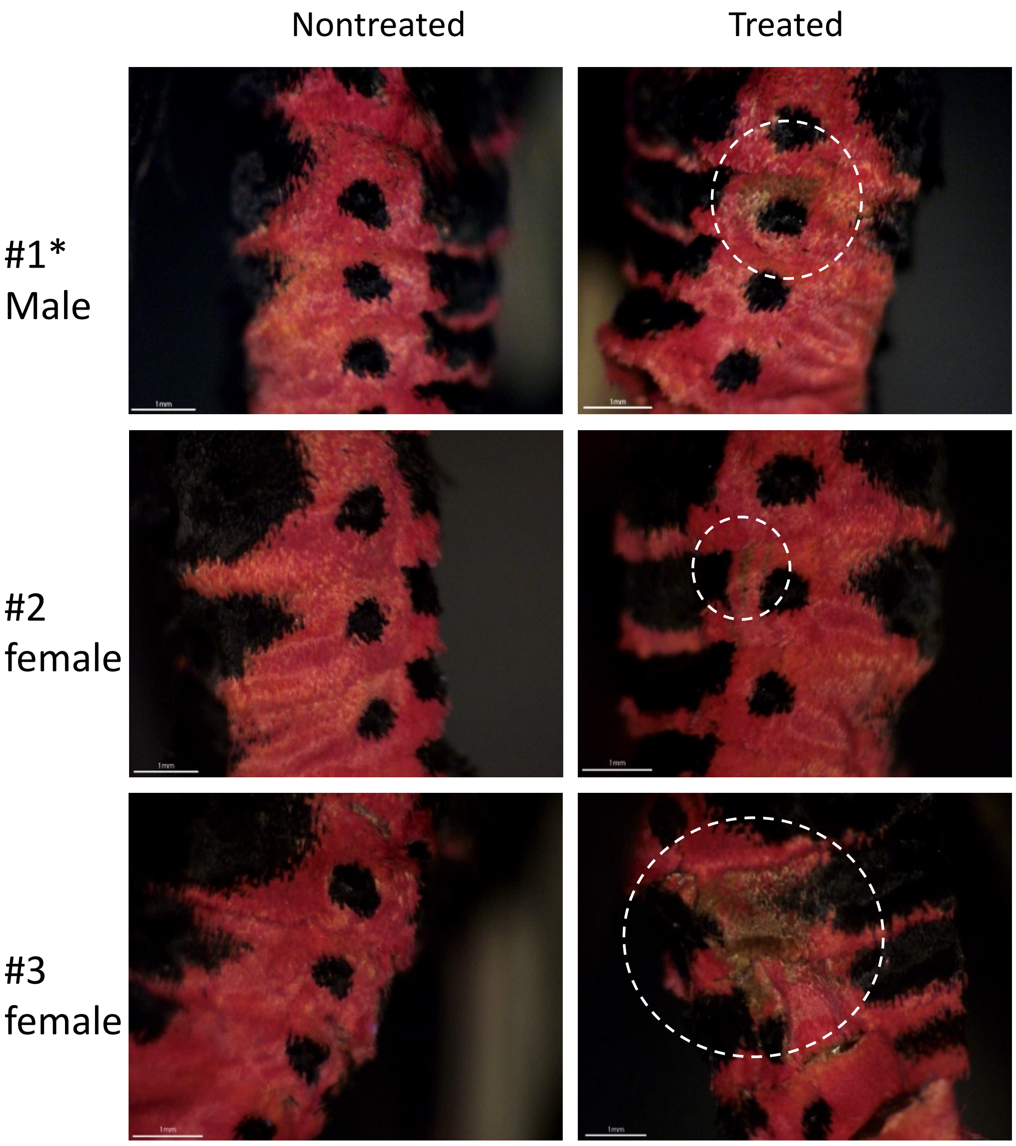

Supplementary Figure S17. Knockdown of *ebony* in the abdomen of *Pachliopta aristolochiae*. Other replicates of Fig. 4f. *#1 is the same individual as Fig. 4f. White dotted circles indicate areas where the phenotypic changes were observed.

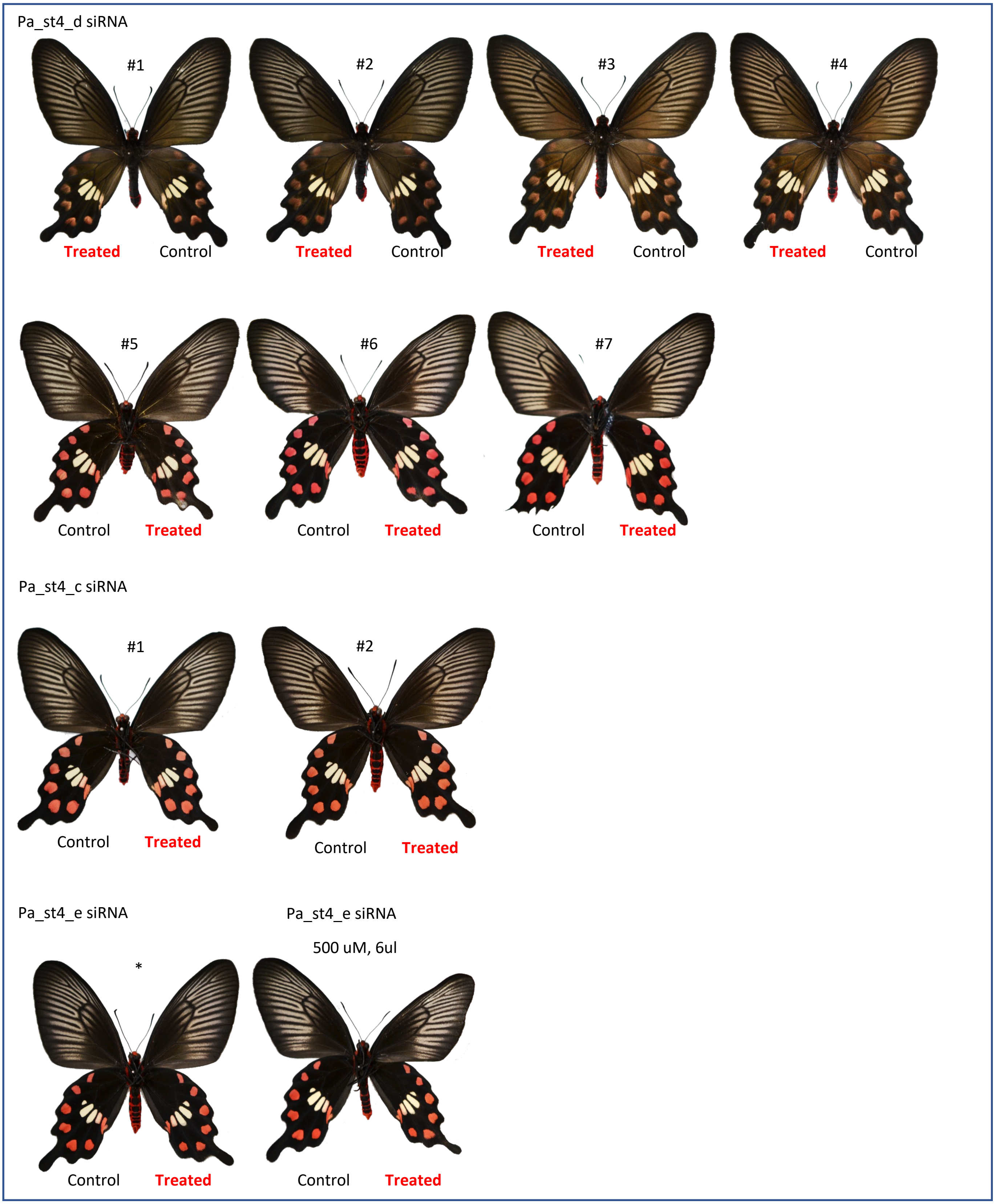

Supplementary Figure S18. Knockdown of *sulfotrasferase4* in the hindwings of *Pachliopta aristolochiae*. Other replicates of Fig. 4h. * is the same individual as Fig. 4h.
