# Supplementary material for: Whole genome analysis of toxic Papilionidae butterflies utilizing aristolochic acid, Pachliopta aristolochiae, and Byasa alcinous
Source: DNA Res. 2025 Dec 22;33(1):dsaf038. doi: 10.1093/dnares/dsaf038 (PMC12784068; doi:10.1093/dnares/dsaf038)
Supplement: dsaf038_Supplementary_Data [file dsaf038_supplementary_data.zip › PaBa_sup_table_DNAres.docx]

**Supplementary Table S1. Statistics of genome sequence reads used for *de novo* genome assembly.**

| Species | Library | Raw read | | Trimmed* | |
| --- | --- | --- | --- | --- | --- |
|  |  | Total (Gbp) | Mean read length (bp) | Total (Gbp) | Mean read length (bp) |
| *Pachiliopta aristlochiae* | Paired-end | 33.61 | 250 | 31.45 | 234 |
|  | Mate-pair 3k | 4.30 | 100 | 2.80 | 87 |
|  | Mate-pair 6k | 3.99 | 100 | 2.62 | 86 |
|  | Mate-pair 10k | 4.40 | 100 | 2.90 | 87 |
|  | Mate-pair 15k | 4.37 | 100 | 2.91 | 87 |
|  |  |  |  |  |  |
| *Byasa alcinous* | Paired-end | 41.65 | 250 | 39.92 | 240 |
|  | Mate-pair 3k | 5.49 | 100 | 3.42 | 86 |
|  | Mate-pair 6k | 5.73 | 100 | 3.42 | 85 |
|  | Mate-pair 10k | 5.63 | 100 | 3.29 | 84 |
|  | Mate-pair 15k | 5.68 | 100 | 3.16 | 83 |

* Trimmed by Platanus_trim (v1.0.7)

**Supplementary Table S2. Summary of RNA-seq data used in this study.**

| Species | Sex | Stage* | Tissue | Total reads | Accession number | Usage |
| --- | --- | --- | --- | --- | --- | --- |
| *Pachliopta aristolochiae* | female | W | hind wing | 33,759,322 | DRR614393 | Gene annotation and expression analysis |
|  | female | W | hind wing | 22,584,948 | DRR614394 |  |
|  | female | W | hind wing | 20,917,962 | DRR614395 |  |
|  | female | P0 | hind wing | 31,412,322 | DRR614396 |  |
|  | female | P0 | hind wing | 46,109,492 | DRR614397 |  |
|  | female | P0 | hind wing | 31,260,080 | DRR614398 |  |
|  | female | P7 | hind wing | 32,238,522 | DRR614399 |  |
|  | female | P7 | hind wing | 23,875,268 | DRR614400 |  |
|  | female | P7 | hind wing | 25,632,622 | DRR614401 |  |
|  | female | P7 | hind wing | 50,731,176 | DRR614402 |  |
|  | female | P13 | hind wing | 46,511,134 | DRR614403 |  |
|  | female | P13 | hind wing | 57,185,956 | DRR614404 |  |
|  | female | P13 | hind wing | 45,178,016 | DRR614405 |  |
|  | female | P14 | hind wing  (red region) | 35,156,558 | DRR614406 | DEGs analysis between red and black wing regions |
|  | female |  | hind wing  (red region) | 28,744,490 | DRR614407 |  |
|  | female |  | hind wing  (red region) | 30,440,134 | DRR614408 |  |
|  | female |  | hind wing (black region) | 34,229,552 | DRR614409 |  |
|  | female |  | hind wing (black region) | 18,322,720 | DRR614410 |  |
|  | female |  | hind wing (black region) | 27,388,204 | DRR614411 |  |
|  | - | 5^th^ instar larvae | mid gut | 61,421,596 | DRR614412 | Expression analysis |
|  | - |  | mid gut | 45,496,040 | DRR614413 |  |
|  | - |  | mid gut | 45,098,992 | DRR614419 |  |
| *Byasa alcinous* | - | 1^st^ instar larvae | epithelium (flat, 0hr after hatching) | 46,435,304 | DRR614393 | Gene annotation |
|  | - |  |  | 22,810,370 | DRR614394 |  |
|  | - |  |  | 37,401,304 | DRR614395 |  |
|  | - |  | epithelium (protrusion, 0hr after  hatching) | 34,430,312 | DRR614396 |  |
|  | - |  |  | 24,053,492 | DRR614397 |  |
|  | - |  |  | 27,428,512 | DRR614398 |  |
|  | - |  | epithelium (flat, 15hr after hatching) | 30,197,508 | DRR614399 |  |
|  | - |  |  | 39,049,750 | DRR614400 |  |
|  | - |  |  | 87,319,248 | DRR614401 |  |
|  | - |  | epithelium (protrusion, 15hr after hatching) | 40,765,408 | DRR614402 |  |
|  | - |  |  | 32,858,772 | DRR614403 |  |
|  | - |  |  | 20,770,834 | DRR614404 |  |
|  | - | P0 | hind wing | 63,112,080 | DRR614405 | Expression analysis |
|  | - | P0 | hind wing | 57,318,592 | DRR614406 |  |
|  | - | P0 | hind wing | 48,500,848 | DRR614407 |  |
|  | - | P2 | hind wing | 50,383,290 | DRR614408 |  |
|  | - | P2 | hind wing | 53,295,846 | DRR614409 |  |
|  | - | P2 | hind wing | 55,918,344 | DRR614410 |  |

**Supplementary Table S3. Statistics of draft genome**. The draft genomes of *Pachliopta aristolochiae* and *Byasa alcinous* are constructed in this study and draft genomes of other butterflies and moths are included for comparison.

|  | Total (bp) | Scaffold N50 (bp) | Scaffold L50 (#) | Contig N50 (bp) | Contig L50 (#) | Gap rate (%) | Reference |
| --- | --- | --- | --- | --- | --- | --- | --- |
| ***Pachliopta aristlochiae*** | **312,133,182** | **7,834,959** | **15** | **238.3K** | **388** | **1.38** | **This study** |
| ***Byasa alcinous*** | **257,647,964** | **9,455,353** | **10** | **252.7K** | **286** | **0.51** | **This study** |
| *Battus philenor* | 443,215,375 | 15,242,119 | 14 | 14.6M | 14 | 0.0002 | NCBI RefSeq  (GCF_028537555.1) |
| *Papilio memnon* | 232,545,187 | 5,456,730 | 16 | 12.412K | 4482 | 5.72 | Iijima et al. 2018 |
| *Papilio polytes* | 227,021,014 | 3,672,263 | 21 | 48.813K | 1,111 | 3.85 | NCBI RefSeq (GCF_000836215.1) |
| *Papilio bianor* | 421,524,737 | 13,120,256 | 15 | 3.106M | 41 | 0.07 | Lu et al. 2019 |
| *Papilio machaon* | 252,114,018 | 8,777,606 | 13 | 8.6M | 13 | 0.00 | NCBI RefSeq (GCF_912999745.1) |
| *Papilio xuthus* | 223,546,057 | 6,417,446 | 14 | 142.71K | 452 | 1.90 | NCBI RefSeq (GCF_000836235.2) |
| *Iphiclides podalirius* | 430,729,501 | 15,090,856 | 13 | 5.184M | 26 | 0.0021 | Mackintosh et al. 2022 |
| *Danaus plexippus* | 245,173,502 | 8,158,176 | 13 | 3.941M | 21 | 0.002 | NCBI RefSeq  (GCF_018135715.1) |
| *Bombyx mori* | 461,704,601 | 16,894,347 | 13 | 16M | 13 | 0.37 | NCBI RefSeq  (GCF_030269925.1) |

**Supplementary Table S4. BUSCO (v5.4.3) statistics with lepidoptera_odb10 database (n=5286) of the genome assemblies.** As Supplementary table S2, the draft genomes of *Pachliopta aristolochiae* and *Byasa alcinous* are constructed in this study and draft genomes of other butterflies and moth are cited from previous studies. See the references in Supplementary Table S3.

|  | Complete (%) | Single complete (%) | Duplicated complete (%) | Fragmented (%) | Missing (%) |
| --- | --- | --- | --- | --- | --- |
| ***P. aristlochiae*** | **98.9** | **98.6** | **0.3** | **0.2** | **0.9** |
| ***B. alcinous*** | **98.9** | **98.8** | **0.1** | **0.3** | **0.8** |
| *Battus philenor* | **98.7** | **98.4** | **0.3** | **0.3** | **1.0** |
| *Papilio memnon* | 98.3 | 97.5 | 0.8 | 0.7 | 1.0 |
| *Papilio polytes* | 95.9 | 95.7 | 0.2 | 1.6 | 2.5 |
| *Papilio bianor* | 97.0 | 96.7 | 0.3 | 0.5 | 2.5 |
| *Papilio machaon* | 99.3 | 98.3 | 1.0 | 0.1 | 0.6 |
| *Papilio xuthus* | 99.1 | 98.9 | 0.2 | 0.2 | 0.7 |
| *Iphiclides podalirius* | 96.7 | 96.5 | 0.2 | 0.4 | 2.9 |
| *Danaus plexippus* | 98.4 | 97.9 | 0.5 | 0.6 | 1.0 |
| *Bombyx mori* | 98.7 | 98.5 | 0.2 | 0.4 | 0.9 |

**Supplementary Table S5. Statistics of protein-coding genes.** *Pachliopta aristolochiae* and *Byasa alcinous* are annotated in this study. The other were annotated in the previous studies (see the references in Supplementary Table S3).

| Species | #genes | total gene length | mean gene length | #exons | mean exons/mrna | #single exon gene | total exon length | mean exon length | mean cds length | #intron | total intron length | mean intron length |
| --- | --- | --- | --- | --- | --- | --- | --- | --- | --- | --- | --- | --- |
| ***Pachliopta aristlochiae*** | **13,497** | **148,218,701** | **10,981** | **98,324** | **7.3** | **1,337** | **21,154,037** | **215** | **1,567** | **84,827** | **127,064,664** | **1,497** |
| ***Byasa alcinous*** | **14,669** | **129,124,723** | **8,802** | **101,483** | **6.9** | **1,551** | **21,664,960** | **213** | **1,476** | **86,814** | **107,459,763** | **1,237** |
| *Battus philenor* | 12,266 | 198,290,910 | 16166 | 93,841 | 7.7 | 1,389 | 20,382,507 | 217 | 1,661 | 81,575 | 177,908,403 | 2,181 |
| *Papilio memnon* | 12,421 | 126,481,468 | 10,182 | 91,275 | 7.3 | 1,286 | 26,310,344 | 288 | 1,508 | 74,085 | 84,748,350 | 1,143 |
| *Papilio polytes* | 12,178 | 134,725,154 | 11,062 | 140,299 | 8.4 | 923 | 37,906,595 | 270 | 1,631 | 117,262 | 154,479,641 | 1,317 |
| *Papilio bianor* | 15,375 | 136,940,518 | 8,906 | 98,231 | 6.4 | 1,013 | 21,270,770 | 216 | 1,383 | 82,856 | 115,669,748 | 1,396 |
| *Papilio machaon* | 13,885 | 297,336,464 | 21,414 | 282,435 | 10.3 | 1,289 | 89,236,500 | 315 | 2,071 | 240,882 | 627,009,358 | 2,602 |
| *Papilio xuthus* | 12,804 | 141,220,227 | 11,029 | 218,745 | 10.2 | 1,111 | 61,997,998 | 283 | 1,936 | 187,044 | 213,006,769 | 1,138 |
| *Iphiclides podalirius* | 17,817 | 148,625,219 | 8,341 | 105,210 | 5.9 | 2,827 | 19,722,463 | 187 | 1,106 | 87,393 | 128,383,861 | 1,469 |
| *Danaus plexippus* | 15,692 | 115,478,003 | 7,359 | 91,286 | 5.8 | 2,643 | 19,081,428 | 209 | 1,215 | 75,594 | 96,396,575 | 1,275 |
| *Bombyx mori* | 13,455 | 216,887,043 | 16,119 | 97,901 | 7.3 | 1,725 | 22,569,794 | 231 | 1,677 | 84,446 | 194,317,249 | 2301 |

**Supplementary Table S6. BUSCO (v5.4.3) statistics with lepidoptera_odb10 database (n=5286) of the gene annotation.** *Pachliopta aristolochiae* and *Byasa alcinous* are annotated in this study. The other were annotated in the previous studies (see the references in Supplementary Table S3).

|  | Complete (%) | Single complete (%) | Duplicated complete (%) | Fragmented (%) | Missing (%) |
| --- | --- | --- | --- | --- | --- |
| ***Pachliopta aristlochiae*** | **98.2** | **97.9** | **0.3** | **0.2** | **1.6** |
| ***Byasa alcinous*** | **98.9** | **98.5** | **0.4** | **0.2** | **0.9** |
| *Battus philenor* | 98.9 | 98.5 | 0.4 | 0.1 | 1.0 |
| *Papilio memnon* | 88.8 | 86.0 | 2.8 | 1.8 | 9.4 |
| *Papilio polytes* | 96.4 | 96.1 | 0.3 | 1.8 | 1.8 |
| *Papilio bianor* | 90.9 | 90.3 | 0.6 | 2.0 | 7.1 |
| *Papilio machaon* | 99.6 | 98.7 | 0.9 | 0.1 | 0.3 |
| *Papilio xuthus* | 99.6 | 99.5 | 0.1 | 0.1 | 0.3 |
| *Iphiclides podalirius* | 79.6 | 78.8 | 0.8 | 5.5 | 14.9 |
| *Danaus plexippus* | 99.4 | 97.6 | 1.8 | 0.1 | 0.5 |
| *Bombyx mori* | 98.3 | 97.9 | 0.4 | 0.2 | 1.5 |

**Supplementary Table S7. Significantly expand gene groups in *P*. *aristolochiae* and *B*. *alcinous* estimated by Sonicparanoid2 and cafe5. (Excel file)**

**Supplementary Table S8. Significantly contracted gene groups in *P. aristolochiae* and *B. alcinous* estimated by Sonicparanoid2 and cafe5. (Excel file)**

**Table S9. Positive selection in single-copy genes estimated by Sonicparanpid and PAML(codeml). (Excel file)**

**Supplementary Table S10. Genes highly expressed in red spots compared to black regions in *P. aristolochiae* (Excel file).**

**Supplementary Table S11. List of siRNA**

| siRNA target gene | siRNA name | Sense (5'–3') | Antisense (5'–3') |
| --- | --- | --- | --- |
| *TH* | PaTH_A | CAAGUACGGCGACUCAAUUCC | AAUUGAGUCGCCGUACUUGUA |
| *ebony* | Pa_ebony_A | GAAAUUGGUUCGGAUUCUAGC | UAGAAUCCGAACCAAUUUCUG |
| *black* | Pablack_A | GCAUACACUCCUUUACUCUUC | AGAGUAAAGGAGUGUAUGCAG |
| *laccase2* | Palaccase2_A | CCAACAACGGCUAUGUUGUAU | ACAACAUAGCCGUUGUUGGGC |
| *Sulfotransferase 4* | Pa_st4_d | GGAUGUAGCGGUCUCUUUUUA | AAAAGAGACCGCUACAUCCCU |
| *Sulfotransferase 4* | Pa_st4_c | GGGCCCUAAGGGUUACUUUUU | AAAGUAACCCUUAGGGCCCAC |
| *Sulfotransferase 4* | Pa_st4_e | CCUUGAUACAGCUAAAGUUGU | AACUUUAGCUGUAUCAAGGAG |

**Supplementary Table S12. Common genes in the list of genes in the expanded gene groups (EGGs) and the list of highly expressed genes (HEGs) in the red spot of hindwings**

| P.aristolochiaeGene00772 | XP_012547171.1\|transmembrane protease serine 9\|0.0\|569 | cocoonase (Coc) | g196 |
| --- | --- | --- | --- |
| P.aristolochiaeGene02346 | XP_004921941.1\|glucose dehydrogenase\|0.0\|1008 | ecdysone oxidase-like | g135 |
| P.aristolochiaeGene02347 | XP_028043823.1\|LOW QUALITY PROTEIN: glucose dehydrogenase\|0.0\|999 | ecdysone oxidase-like | g135 |
| P.aristolochiaeGene02351 | XP_028043823.1\|LOW QUALITY PROTEIN: glucose dehydrogenase\|0.0\|999 | ecdysone oxidase-like | g135 |
| P.aristolochiaeGene04165 | XP_012547486.2\|facilitated trehalose transporter Tret1\|0.0\|1008 | facilitated trehalose transporter Tret1-2 homolog | g50 |
| P.aristolochiaeGene04551 | XP_012553399.1\|uncharacterized protein LOC105843019\|8e-177\|494 | uncharacterized LOC101744925 | g14 |
| P.aristolochiaeGene05397 | XP_004933321.1\|aldo-keto reductase AKR2E4-like\|0.0\|707 | aldo-keto reductase AKR2E4-like | g186 |
| P.aristolochiaeGene09812 | XP_004923009.1\|protein takeout\|3e-177\|496 | protein takeout | g17 |
| P.aristolochiaeGene10185 | NP_001103386.1\|cocoonase precursor\|0.0\|532 | cocoonase (Coc) | g196 |
| P.aristolochiaeGene10186 | NP_001103386.1\|cocoonase precursor\|0.0\|532 | cocoonase (Coc) | g196 |

**Supplementary Table S13. Genes common in the list of genes undergoing positive selection (PSGs) and in the list of highly expressed genes (HEGs) in the red spot of hindwings**

| P.aristolochiaeGene01366 | XP_028034984.1\|uncharacterized protein LOC114246586 isoform X3\|0.0\|3577 | |
| --- | --- | --- |
| P.aristolochiaeGene01798 | XP_028031989.1\|indole-3-acetaldehyde oxidase-like isoform X3\|0.0\|2590 | |
| P.aristolochiaeGene02390 | XP_028028039.1\|uncharacterized protein LOC114241409\|0.0\|1831 | |
| P.aristolochiaeGene02612 | XP_028025472.1\|retinal-specific ATP-binding cassette transporter-like\|0.0\|2049 | |
| P.aristolochiaeGene03009 | XP_004929730.1\|organic cation transporter protein\|0.0\|1103 | |
| P.aristolochiaeGene04040 | XP_004932075.1\|sialin\|0.0\|1056 | |
| P.aristolochiaeGene04940 | XP_012552272.2\|cilia- and flagella-associated protein 58\|6e-124\|373 | |
| P.aristolochiaeGene05433 | XP_004924995.1\|protein seele isoform X1\|2e-180\|503 | |
| P.aristolochiaeGene05822 | AJQ30182.1\|aspartate decarboxylase\|0.0\|1064 | |
| P.aristolochiaeGene05844 | XP_021208081.1\|MATH and LRR domain-containing protein PFE0570w\|0.0\|2090 | |
| P.aristolochiaeGene05935 | XP_028028320.1\|protein scarlet-like isoform X2\|0.0\|929 | |
| P.aristolochiaeGene06357 | XP_012551219.2\|melanization protease 1-like isoform X1\|0.0\|952 | |
| P.aristolochiaeGene06426 | NP_001037478.1\|p270\|0.0\|3959 | |
| P.aristolochiaeGene06428 | XP_004928951.1\|limulus clotting factor C isoform X2\|0.0\|1234 | |
| P.aristolochiaeGene06896 | XP_012550840.2\|probable serine/threonine-protein kinase tsuA isoform X1\|0.0\|1443 | |
| P.aristolochiaeGene06931 | BAC57964.1\|calreticulin\|0.0\|796 | |
| P.aristolochiaeGene08334 | FAA00644.1\|TPA: putative cuticle protein\|0.0\|895 | |
| P.aristolochiaeGene08516 | XP_028036000.1\|uncharacterized protein LOC114247282 isoform X1\|0.0\|1899 | |
| P.aristolochiaeGene09545 | XP_028026449.1\|echinoderm microtubule-associated protein-like 2 isoform X2\|0.0\|1278 | |
| P.aristolochiaeGene09551 | XP_028039670.1\|V-type proton ATPase catalytic subunit A\|0.0\|1271 | |
| P.aristolochiaeGene10293 | XP_012547180.1\|lipase member H-B\|0.0\|672 | |
| P.aristolochiaeGene10673 | XP_021205684.1\|uncharacterized protein LOC105841811 isoform X1\|0.0\|1206 | |
| P.aristolochiaeGene10763 | XP_004922817.1\|prostatic acid phosphatase isoform X1\|0.0\|803 | |
| P.aristolochiaeGene10844 | XP_021209323.1\|solute carrier family 12 member 9 isoform X2\|0.0\|1677 | |
| P.aristolochiaeGene10894 | XP_012549806.1\|P protein isoform X1\|0.0\|1391 | |
| P.aristolochiaeGene11149 | XP_012548461.1\|endonuclease/exonuclease/phosphatase family domain-containing protein 1\|0.0\|1133 | |
| P.aristolochiaeGene11371 | XP_028029069.1\|protein cueball isoform X2\|0.0\|889 | |
| P.aristolochiaeGene12587 | XP_004927105.1\|uncharacterized protein LOC101745285\|0.0\|650 |  |
| P.aristolochiaeGene12596 | XP_004921981.1\|mesencephalic astrocyte-derived neurotrophic factor homolog\|5e-120\|345 |  |
